# Enhanced mitochondrial fission promotes chemosensitivity via SHP-1 activity in triple-negative breast cancer

**DOI:** 10.1007/s11033-026-12615-y

**Published:** 2026-08-25

**Authors:** Elizabeth K. Croom, Lillian E. Walton, Alessandro J. Bono, Anne Zhang, Brock A. Humphries

**Affiliations:** Center for Molecular Imaging, Department of Radiology, University of Michigan, 109 Zina Pitcher Place, Ann Arbor, MI 48109 USA

**Keywords:** Triple-negative breast cancer, Mitochondrial dynamics, Drug resistance, SHP-1 (PTPN6), SHP-2 (PTPN11), Cell signaling

## Abstract

**Background:**

Triple-negative breast cancer (TNBC) is an aggressive subtype with high chemoresistance and poor survival rates. While mitochondrial dynamics, fission and fusion, are implicated in chemoresistance, their precise roles remain largely unexplored. This study investigates how cytoplasmic phosphatases SHP-1 and SHP-2 regulate chemotherapeutic response in TNBC cells with altered mitochondrial dynamics.

**Methods:**

We utilized click beetle green luciferase-expressing SUM149 and SUM159 TNBC cells engineered to stably express mitochondrial dynamics modulators (PISD, Drp1, MFN2). SHP-1/2 activity was further manipulated using either the inhibitor NSC87877 or stable shRNA knockdown, confirmed by qRT-PCR and Western Blotting. Cell growth and cytotoxicity were evaluated through bioluminescence imaging and drug assays using doxorubicin, paclitaxel, and salinomycin.

**Results:**

We show that SUM149 and SUM159 TNBC cells engineered to exhibit enhanced mitochondrial fission via increased PISD and Drp1 expression are more sensitive to doxorubicin and salinomycin, but not paclitaxel. SHP-1/2 inhibition attenuated doxorubicin and salinomycin sensitivity specifically in mitochondrial fission-enriched cells, with minimal effects in fusion-enriched cells and no effect on paclitaxel response. Notably, only SHP-1 knockdown reduced drug sensitivity in cells with enhanced mitochondrial fission, highlighting SHP-1 as a key regulator of chemoresistance. Conversely, enhanced mitochondrial fusion conferred resistance to cytotoxic agents.

**Conclusions:**

Our *in vitro* findings demonstrate that mitochondrial fission sensitizes TNBC cells to certain chemotherapeutics, and that SHP-1 critically regulates this response. This newly identified mechanism links mitochondrial dynamics to chemotherapeutic sensitivity, offering a potential pathway to overcome TNBC chemoresistance through targeted therapies.

**Supplementary Information:**

The online version contains supplementary material available at 10.1007/s11033-026-12615-y.

## Introduction

Triple-negative breast cancer (TNBC) is the second most common subtype of breast cancer, and despite advances in therapy, patients with TNBC have the poorest overall survival. Patients with TNBC experience higher rates of local and distant recurrence compared to other subtypes of breast cancer, including those with small tumors [1]. Although many TNBC tumors initially respond to first-line chemotherapeutics, treatment often unintentionally selects for pre-existing chemoresistant genotypes which result in frequent relapse [2]. Therefore, a deeper understanding of the molecular mechanisms driving chemoresistance is needed to identify new therapeutic targets and enhance the effectiveness of current treatments.

One hallmark of cancer is the reprogramming of cellular metabolism to promote survival under stress, often involving shifts in mitochondrial energetics [3, 4]. Mitochondria exhibit a dynamic balance between fission and fusion (mitochondrial dynamics), which directly influences cellular metabolism and can affect cellular responses to their environment [5, 6]. Not only do cancer cells remodel their mitochondria to fuel growth and survival in response to their environment, but work has shown that mitochondria can modulate chemoresistance through various mechanisms [7–9]. However, the precise relationship between mitochondrial dynamics and chemoresistance is not thoroughly appreciated.

Among regulators of cell signaling, the protein-tyrosine phosphatase (PTP) superfamily is notable for modulating critical processes in tumor biology, including proliferation, apoptosis, and response to therapy. In this context, the closely related cytoplasmic phosphatases SHP-1 (encoded by *PTPN6*) and SHP-2 (encoded by *PTPN11*) have emerged as particularly interesting candidates for investigation, based on several key lines of evidence. First, both SHP-1 and SHP-2 are activated by reactive oxygen species (ROS)-dependent oxidation [10, 11], a mechanism closely linked to mitochondrial metabolism. Second, although high expression levels of SHP-1/2 have been reported in breast cancer [12, 13], they exert distinct, and sometimes opposing, effects, where SHP-1 generally functions as a tumor suppressor, while SHP-2 promotes oncogenic processes such as proliferation and survival [14–17]. Third, recent studies, including our own work, have shown that SHP-2 localizes to mitochondria and that mitochondrial dynamics can directly regulate the activity of both SHP-1 and SHP-2 [18–21]. This unique positioning at the intersection of mitochondrial signaling and cancer cell fate suggests that SHP-1/2 may be key mediators of mitochondrial dynamics-driven TNBC chemoresistance, which is highlighted by metabolic reprogramming.

To address this gap, we investigated whether altered mitochondrial dynamics regulate TNBC cell responses to select chemotherapeutics, and whether SHP-1/2 phosphatases mediate these effects. Using TNBC cells engineered toward mitochondrial fission or fusion, we examined sensitivity to chemotherapeutic agents with distinct mechanisms of action, allowing us to probe whether mitochondrial dynamics confers broad or selective drug vulnerability. We further dissected the individual contributions of SHP-1 and SHP-2 to drug response using both pharmacologic inhibition and stable shRNA knockdown, enabling us to distinguish shared from phosphatase-specific effects. Together, our findings identify a mitochondrial fission/SHP-1 signaling axis that contributes to chemotherapy response in TNBC cells, with implications for overcoming drug resistance in this aggressive and difficult-to-treat cancer subtype.

## Materials and methods

### Cell culture

We obtained both SUM149 and SUM159 cells as a gift from Dr. Stephen Ethier (The Medical University of South Carolina, Charleston, SC) and cultured cells in F-12 media supplemented with 10% fetal bovine serum (FBS), 1% penicillin/streptomycin (Pen/Strep), 1% GlutaMAX, 5 μg/mL hydrocortisone, and 1 μg/mL insulin. SUM149 cells have been reported as a basal-like/inflammatory TNBC model with BRCA1 deficiency, whereas SUM159 cells have been reported as a mesenchymal/claudin-low TNBC model that is generally BRCA1 wild-type [22]. We authenticated all cells by analysis of short tandem repeats and characterized cells as free of Mycoplasma at the initial passage. We also cultured cells in an anti-mycoplasma prophylactic (2.5 mg/mL). We used all cells within 3 months after resuscitation and maintained all cells at 37°C in a humidified incubator with 5% CO_2_.

### Chemicals and reagents

FBS, Pen/Strep, GlutaMAX, sodium pyruvate, FluoroBrite DMEM (cat: A1896701), and F-12 medium were purchased from Thermo Fisher Scientific (Waltham, MA, USA). Hydrocortisone was purchased from Millipore Sigma (St. Louis, MO, USA; cat: H0888), insulin was purchased from Millipore Sigma (cat: I9278), and Plasmocin was purchased from InvivoGen (San Diego, CA, USA). L-α-lysophosphatidylethanolamine (LPE; Avanti Polar Lipids Inc., Alabaster, AL, USA; cat: 860081) was used at 50 μM to promote mitochondrial fragmentation. Leflunomide (Lef; Cayman Chemical Company, Ann Arbor, MI, USA; cat: 14860) was used at 50 μM to promote mitochondrial fusion. NSC87877, a pan SHP-1/2 inhibitor, was purchased from Cayman Chemical Company (cat: 14908) and used at 25 μM. Doxorubicin was obtained from Pfizer (New York, NY, USA), salinomycin was purchased from Cayman Chemical Company (cat: 13579), and paclitaxel was obtained from Athenex (Buffalo, NY, USA). MitoTracker Green (cat: M7514) was purchased from Thermo Fisher Scientific and used according to the manufacturer’s instructions.

### Vectors and cell lines

We used SUM149 and SUM159 cells that stably express click beetle green (CBG) luciferase [23], as well as phosphatidylserine decarboxylase (PISD), dynamin related protein 1 (Drp1), or mitofusin 2 (MFN2) to enforce mitochondrial morphologies as described in our previous publications [21, 24].

We purchased shRNA constructs targeting human SHP1 (PTPN6, hairpin number TRCN0000244305) and SHP2 (PTPN11, hairpin number TRCN0000005003) from Millipore Sigma through the University of Michigan Vector Core. We purchased non-target shRNA control construct directly from Millipore Sigma (cat: SHC016). We prepped shRNA constructs and produced lentivirus as described previously [25]. We generated WT control, PISD, Drp1, and MFN2 cells that stably expressed control and shRNA constructs as described previously through selection with puromycin [23]. We confirmed knockdown of SHP1 and SHP2 in these cells through qRT-PCR.

### Fluorescence microscopy

We visualized mitochondrial morphology in live cells as described previously [24]. Briefly, we seeded 2 × 10^4^ cells onto 35-mm dishes with a 20-mm glass bottom insert (Cellvis, Mountain View, CA, USA) in FluoroBrite DMEM media, containing 10% FBS, 1% Pen/Strep, 1% GlutaMAX, 1% Sodium Pyruvate, 5 μg/mL hydrocortisone, and 1 μg/mL insulin. Two and three days after seeding, we changed media to fresh FluoroBrite DMEM as described above containing either l-α-lysophosphatidylethanolamine (LPE; 50 μM) to drive fragmentation, leflunomide (Lef; 50 μM) to drive fusion, or vehicle control. The next day (four days post seeding) we stained cells with MitoTracker Green following the manufacturer’s instructions. We acquired all images on an Olympus IX73 microscope with a DP80 CCD camera using cellSens software (Olympus). We analyzed mitochondrial morphology as described in our previous publications [21, 24]. Briefly, we used MATLAB to skeletonize mitochondria and measure total pixel area and major axis length for all mitochondria within an image. We separated mitochondria into fused or fissioned groups by area and major axis length using a user-defined cutoff that we applied to every cell group analyzed in each experiment. For each identified mitochondrial object, we calculated an empirical, pixel-based area-to-length metric by dividing total mitochondrial pixel area by major axis length, using MATLAB’s regionprops command. We categorized an identified mitochondrion that had total area/majoraxislength >2, in this pixel-based metric, as networked and did not include these mitochondria in the analysis.

### Bioluminescence imaging

For growth curves, we seeded 1 x 10^3^ cells per well in a black-walled 96 well plate (Corning, Kennebunk, ME, USA, cat: 3603). We changed media to complete F12 media supplemented as described above the day after seeding. For each day shown in the figure legend, we performed bioluminescence imaging of CBG using an IVIS Lumina Series III (Perkin Elmer, Waltham, MA, USA) as previously described [23]. We exported photon flux data from regions of interest and quantified changes in viability in Living Image 4.3.1 (Perkin Elmer).

For cytotoxicity curves utilizing LPE and Lef, we initially seeded 2 × 10^4^ cells onto 35-mm dishes in FluoroBrite DMEM media, containing 10% FBS, 1% Pen/Strep, 1% GlutaMAX, and 1% Sodium Pyruvate as described previously [24]. Two and 3 days after seeding, we changed media to FluoroBrite DMEM media as described above containing either LPE (50 μM) to drive fragmentation, leflunomide to drive fusion, or vehicle control. The next day, four days after seeding, these cells were then used for cytotoxicity curves.

For cytotoxicity curves utilizing NSC87877, we initially seeded 2 × 10^4^ cells onto 35-mm dishes in FluoroBrite DMEM media with supplementation as described previously above. Two days after seeding, we changed media to FluoroBrite DMEM media as described above containing NSC87877 (25 µM) or vehicle control [21]. The next day, three days post seeding, we used the cells for cytotoxicity curves.

For all cytotoxicity curves, after treatment as described above we seeded 2 x 10^3^ cells per well of a 96-well plate. The day after seeding, we treated cells with compounds diluted in media at concentrations shown in figures. We used doxorubicin, salinomycin, and paclitaxel. Three days after treatment with concentrations shown in figures, we performed bioluminescence imaging of CBG using an IVIS Lumina Series III (Perkin Elmer) as previously described [23]. We exported photon flux data from regions of interest and quantified changes in viability in Living Image 4.3.1 (Perkin Elmer).

### qRT-PCR

To analyze levels of SHP1 and SHP2, we performed qRT-PCR for SHP1, SHP2, and β-actin using SYBR Green detection as described previously [24]. Unless otherwise stated, we used and combined data from two unique sets of predesigned KiCqStart SYBR Green primers (Millipore Sigma) for the following genes:

PTPN6_1: 5’-CAAGAACATTCTCCCCTTTG-3’ and 5’-TCTTGATGTAGTTGGCATTG-3’

PTPN6_3: 5′-GTGGAGCATTTCAAGAAGAC-3′ and 5′-CTTGTTCAGTTCCAACACTC-3′

PTPN11_1: 5’-CCTGAATTTGAAACCAAGTG-3’ and 5’-ACATTTACTCTTTCCTCTCTCC-3’

PTPN11_3: 5’-ATGAGCCTGTTTCAGATTAC-3’ and 5’-TTCCTCTCTCCACTTCTTTC-3’

For the following gene, we used a single predesigned KiCqStart SYBR Green primer set (Millipore Sigma):

β-actin: 5′-TGTACGTTGCTATCCAGGCTGTGC-3′ and 5′-CGGTGAGGATCTTCATGAGGTAGTC-3′.

### Western blotting

We lysed cells using RIPA buffer and detected amounts of target proteins in total cell lysates by western blotting as described previously [21]. For mitochondrial morphology proteins, we probed total cell lysates for PISD (ThermoFisher; cat MA5-26861), Drp1 (Cell Signaling Technology, Danvers, MA, USA; cat 8570), p-Drp1^S616^ (Cell Signaling Technology; cat 4494), MFN2 (Cell Signaling Technology; cat 11925), or β-Actin (Cell Signaling Technology; cat 8457) as a loading control. For SHP-1 and SHP-2, we probed total cell lysates for SHP-1 (Abcam, Waltham, MA, USA; cat AB227503) or SHP-2 (Cell Signaling Technology; cat 3752) followed by β-Actin (Cell Signaling Technology) as a loading control. For STAT3, we probed total cell lysates for phospho-STAT3 Tyr705 (Cell Signaling Technology; Cat# 9145) followed by total STAT3 (Cell Signaling Technology; Cat# 4904) as a loading control. We quantified relative intensities of bands with ImageJ (National Institutes of Health, Bethesda, MD, USA). For PISD, Drp1, p-Drp1^S616^, MFN2, SHP-1, and SHP-2, target band intensities were normalized to β-actin intensity from the corresponding lane, and the resulting values were expressed relative to the indicated control condition within each experiment. For STAT3 activation, phospho-STAT3 Tyr705 band intensity was first normalized to total STAT3 intensity for each sample, and the resulting phospho-STAT3/total STAT3 ratio was then normalized to the indicated control condition within each experiment.

### Quantification and statistical analysis

For experiments comparing only two groups, we used a two-tailed, unpaired Student’s *t*-test. For experiments comparing multiple groups, we used one-way or two-way ANOVA where indicated and corrected for multiple comparisons with Tukey’s multiple comparisons test. Given the sample size and experimental design, parametric tests were used as the default statistical approach for independent biological replicate data and comparisons. Data distributions and variance were inspected for obvious deviations before analysis. IC_50_ was calculated using a nonlinear fit and AUC was calculated in GraphPad Prism 10. We considered *p* < 0.05 as statistically significant (∗ = p < 0.05, ∗∗ = p < 0.01, ∗∗∗ = p < 0.001). We prepared line graphs and bar graphs (mean values + SD or SEM as denoted in figure legends) using GraphPad Prism 10.

## Results

### Enhanced mitochondrial fission increases drug sensitivity to doxorubicin and salinomycin

**Fig. 1:**
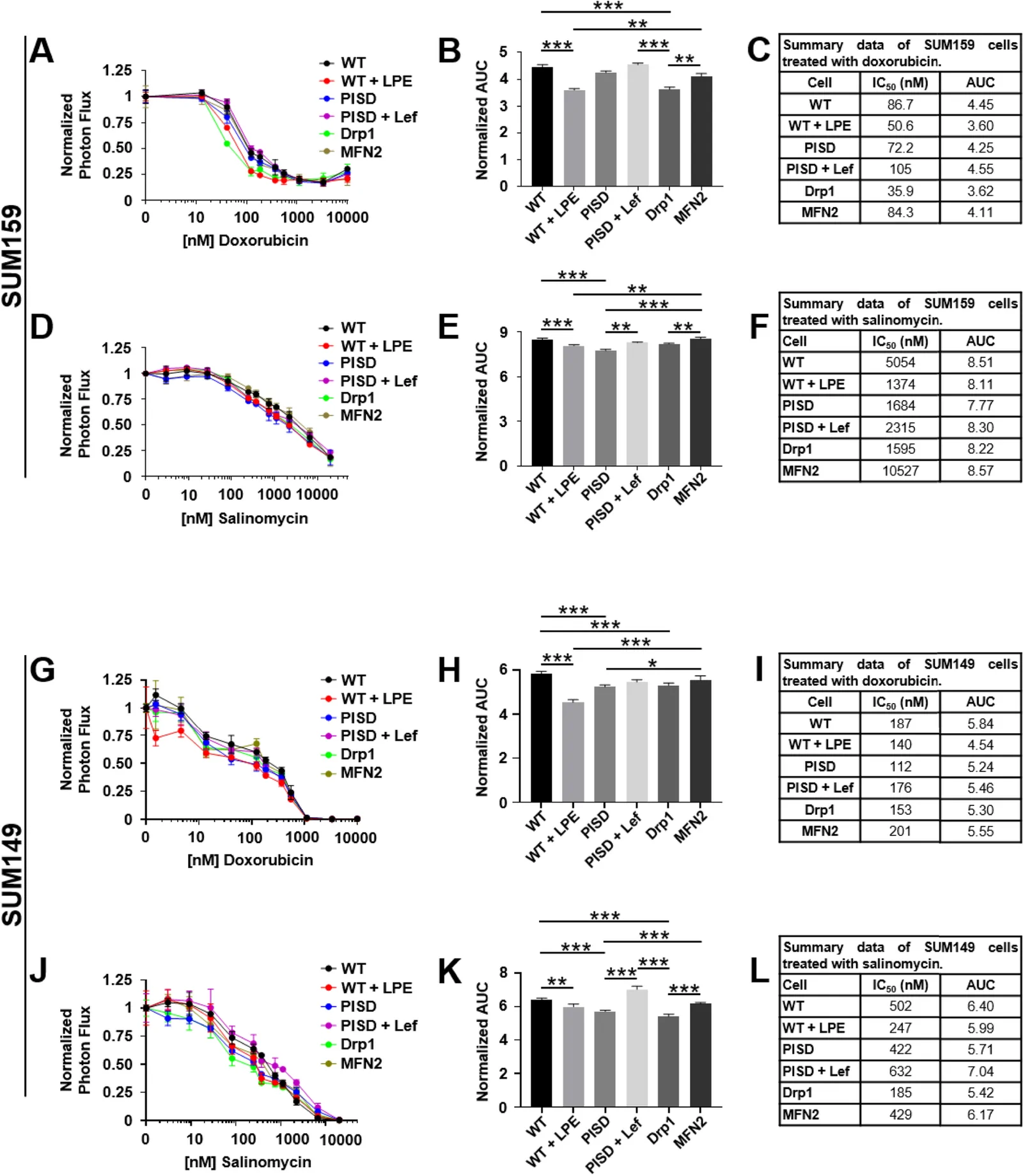
Enhanced mitochondrial fission increases sensitivity to doxorubicin and salinomycin. **A.** Dose-response curves showing mean cellular viability ± SD of SUM159 cells primarily displaying mitochondrial fission, WT + LPE, PISD, and Drp1, or mitochondrial fusion, WT, PISD + Lef, and MFN2, following 72-hour treatment with increasing concentrations of doxorubicin. **B.** Bar graph showing mean area-under-the-curve, AUC, values + SEM normalized to untreated control for the SUM159 doxorubicin response shown in panel A. **C.** Table summarizing IC_50_ and AUC values for SUM159 cells treated with doxorubicin. **D.** Dose-response curves showing mean cellular viability ± SD of SUM159 cells following 72-hour treatment with increasing concentrations of salinomycin. **E.** Bar graph showing mean AUC values + SEM normalized to untreated control for the SUM159 salinomycin response shown in panel D. **F.** Table summarizing IC_50_ and AUC values for SUM159 cells treated with salinomycin. **G.** Dose-response curves showing mean cellular viability ± SD of SUM149 cells following 72-hour treatment with increasing concentrations of doxorubicin. **H.** Bar graph showing mean AUC values + SEM normalized to untreated control for the SUM149 doxorubicin response shown in panel G. **I.** Table summarizing IC_50_ and AUC values for SUM149 cells treated with doxorubicin. **J.** Dose-response curves showing mean cellular viability ± SD of SUM149 cells following 72-hour treatment with increasing concentrations of salinomycin. **K.** Bar graph showing mean AUC values + SEM normalized to untreated control for the SUM149 salinomycin response shown in panel J. **L.** Table summarizing IC_50_ and AUC values for SUM149 cells treated with salinomycin. For dose-response curves, a decrease along the y-axis represents decreased cellular viability relative to untreated control. Error bars for SD are smaller than the symbol for some conditions if not evident. For all panels, n = 4. * = p < 0.05, ** = p < 0.01, *** = p < 0.001. Prioritized comparisons are shown. All comparisons are shown in Supplemental Tables 3-6. Panels A–C, D–F, G–I, and J–L each represent a distinct, independent experiment. Each experiment was repeated at least two additional times (n = 3) with similar results

Although mitochondria have been implicated in drug resistance through their impacts on metabolism or apoptosis [7–9], the impacts of mitochondrial dynamics on drug resistance are much less appreciated. To address this gap in knowledge, we first determined how mitochondrial dynamics contributes to the sensitivity of TNBC cells to two first-line chemotherapeutics, doxorubicin and paclitaxel. We investigated the toxicity of these two standard TNBC therapies using SUM159 and SUM149 TNBC cells that were either wild-type (WT) or engineered to stably express mitochondria morphology-regulating proteins, including PISD, Drp1, and MFN2 **(**Supplemental Fig S1-2 and Supplemental Table 1-2**)** [21, 24]. We found that cells that display enhanced mitochondrial fission (stably expressing PISD or Drp1, or treated with LPE (Supplemental Fig S1)) displayed increased sensitivity to doxorubicin as described by significantly reduced IC_50_ (half-maximum inhibitory concentration) and area-under-curve (AUC, metric that combines potency and efficacy of a drug [26]) in both SUM159 (Fig 1A-C and Supplemental Table 3) and SUM149 (Fig 1G-I and Supplemental Table 4). Conversely, we observed no significant difference in sensitivity between cells that display increased mitochondrial fission versus fusion in treatment with paclitaxel (Supplemental Fig S3).

As TNBC tissues are enriched for inherently drug-resistant cancer stem-like cells [27–29], and differences in mitochondrial dynamics between cancer stem-like cells and bulk cells have been reported [30, 31], we also investigated sensitivity of these cells to salinomycin, a drug that targets cancer stem-like cells [32]. Similar to doxorubicin, we found that cells that display enhanced mitochondrial fission (stably expressing PISD, Drp1, or treated with LPE) displayed increased sensitivity to salinomycin as described by significantly reduced IC_50_ and AUC in both SUM159 (Fig 1D-F and Supplemental Table 5) and SUM149 (Fig 1J-L and Supplemental Table 6).

### Inhibition of SHP-1/2 phosphatases partially recovers drug sensitivity in cells that display mitochondrial fission

**Fig. 2:**
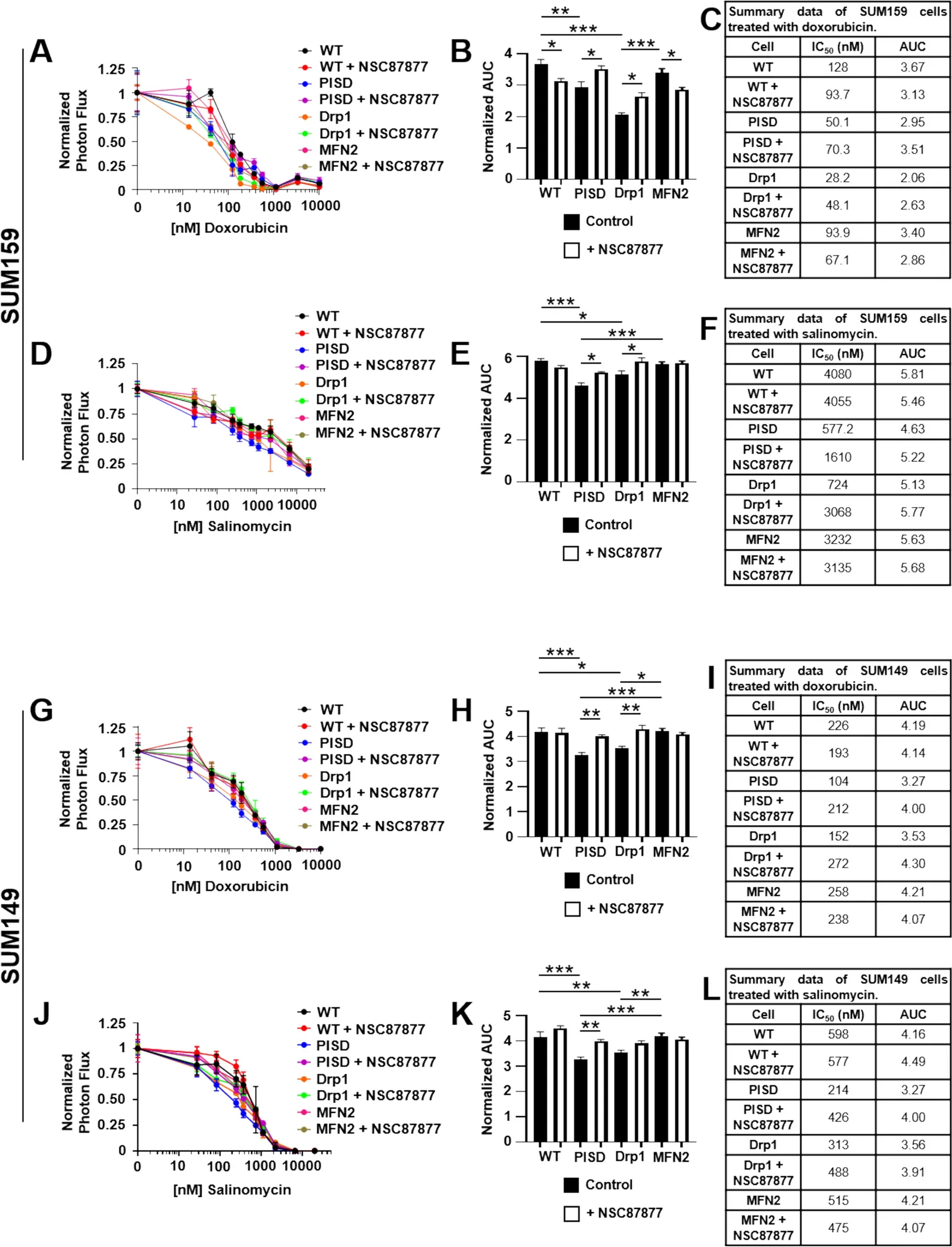
SHP-1/2 inhibition attenuates chemosensitivity in cells with enhanced mitochondrial fission. **A.** Dose-response curves showing mean cellular viability ± SD of SUM159 cells primarily displaying mitochondrial fission, PISD and Drp1, or mitochondrial fusion, WT and MFN2, treated with or without the SHP-1/2 inhibitor NSC87877 and exposed for 72 hours to increasing concentrations of doxorubicin. **B.** Bar graph showing mean area-under-the-curve, AUC, values + SEM normalized to untreated WT control for the SUM159 doxorubicin response shown in panel A. **C.** Table summarizing IC_50_ and AUC values for SUM159 cells treated with doxorubicin. **D.** Dose-response curves showing mean cellular viability ± SD of SUM159 cells treated with or without NSC87877 and exposed for 72 hours to increasing concentrations of salinomycin. **E.** Bar graph showing mean AUC values + SEM normalized to untreated WT control for the SUM159 salinomycin response shown in panel D. **F.** Table summarizing IC_50_ and AUC values for SUM159 cells treated with salinomycin. **G.** Dose-response curves showing mean cellular viability ± SD of SUM149 cells treated with or without NSC87877 and exposed for 72 hours to increasing concentrations of doxorubicin. **H.** Bar graph showing mean AUC values + SEM normalized to untreated WT control for the SUM149 doxorubicin response shown in panel G. **I.** Table summarizing IC_50_ and AUC values for SUM149 cells treated with doxorubicin. **J.** Dose-response curves showing mean cellular viability ± SD of SUM149 cells treated with or without NSC87877 and exposed for 72 hours to increasing concentrations of salinomycin. **K.** Bar graph showing mean AUC values + SEM normalized to untreated WT control for the SUM149 salinomycin response shown in panel J. **L.** Table summarizing IC_50_ and AUC values for SUM149 cells treated with salinomycin. For dose-response curves, a decrease along the y-axis represents decreased cellular viability relative to untreated control. Error bars for SD are smaller than the symbol for some conditions if not evident. For all panels, n = 4. * = p < 0.05, ** = p < 0.01, *** = p < 0.001. Panels A–C, D–F, G–I, and J–L each represent a distinct, independent experiment. Each experiment was repeated at least two additional times (n = 3) with similar results

Our previous work demonstrated that mitochondrial dynamics can impact SHP-1/2 activity to regulate TNBC aggressiveness [21]. Therefore, we expanded upon this data and investigated how inhibition of SHP-1/2 impacts drug resistance in cells that stably display altered mitochondrial dynamics. Similar to our above results, we found that cells that display enhanced mitochondrial fission (PISD and Drp1) displayed increased sensitivity to doxorubicin compared to cells that display enhanced mitochondrial fusion (WT and MFN2) in both SUM159 (Fig 2A-C) and SUM149 (Fig 2G-I). Furthermore, we found that treatment with NSC87877, a SHP-1/2 inhibitor that we found does not impact mitochondrial dynamics [21], significantly decreased doxorubicin sensitivity in cells that display enhanced mitochondrial fission as determined by reduced IC_50_ and AUC. We also found that SHP-1/2 inhibition increased doxorubicin sensitivity in cells that display mitochondrial fusion in SUM159 cells (Fig 2A-C), but not in SUM149 cells. We again observed no significant difference in paclitaxel sensitivity between cells that display increased mitochondrial fission versus fusion or with SHP-1/2 inhibition (Supplemental Fig S4). However, we did find that cells that display enhanced mitochondrial fission are more susceptible to salinomycin. Treatment with NSC87877 decreased sensitivity to salinomycin in cells that display enhanced mitochondrial fission, with little effect in cells that display enhanced fusion in both SUM159 (Fig 2D-F) and SUM149 (Fig 2J-L).

### SHP-1/2 knockdown increases growth in cells with enhanced mitochondrial fission and decreases growth in cells with enhanced fusion

**Fig. 3:**
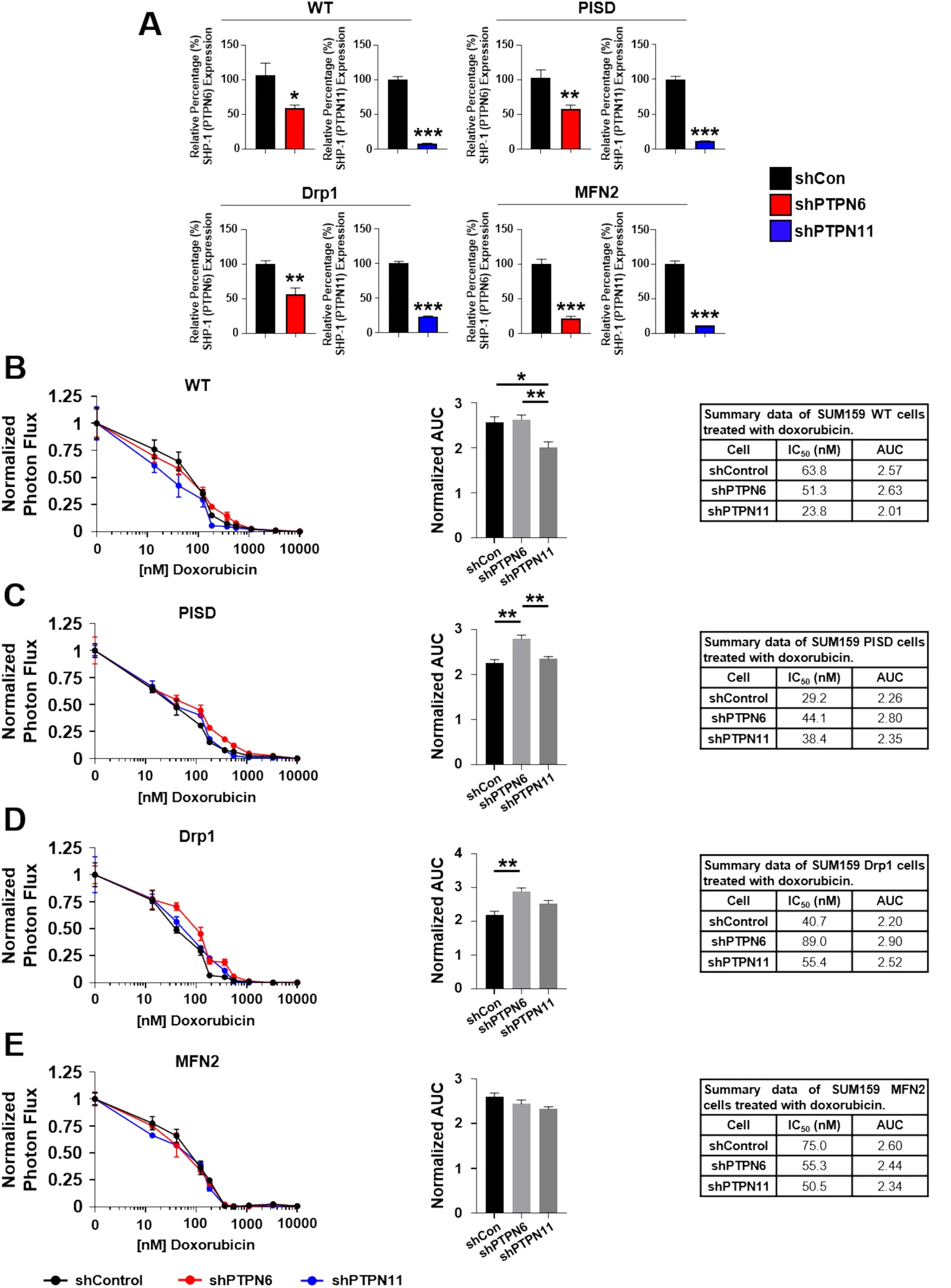
SHP-1 knockdown increases doxorubicin resistance in SUM159 cells with enhanced mitochondrial fission. **A.** qRT-PCR analysis (mean + SEM) of SHP-1 (PTPN6) and SHP-2 (PTPN11) in SUM159 WT, PISD, Drp1, and MFN2 cells (n = 6). Data is relative to SHP-1 and SHP-2 expression in control shRNA (shCon) cells. Data demonstrates that shRNA generates SHP-1 and SHP-2 knockdown in cells. **B-E.** Line graphs show representative mean responses (n = 4) in cellular viability ± SD of SUM159 cells primarily displaying mitochondrial fusion (WT (**B**) and MFN2 (**E**)) or fission (PISD (**C**) and Drp1 (**D**)) with SHP-1 (PTPN6) or SHP-2 (PTPN11) knockdown treated for 72 hours with the commonly used chemotherapeutic, doxorubicin. A decrease along the y-axis represents a decrease in cellular viability versus untreated control. Error bars for SD are smaller than the symbol for some conditions if not evident. Bar graphs demonstrate mean values for area-under-the-curve (AUC) + SEM normalized to untreated control (n = 4) for doxorubicin. Tables on the right summarize the IC_50_ and AUC values for SUM159 cells treated with doxorubicin. All panels represent a distinct, independent experiment. This experiment was repeated at least two other times (n = 3) with similar results. * = *p* < 0.05, ** = *p* < 0.01. *** = *p* < 0.001

**Fig. 4:**
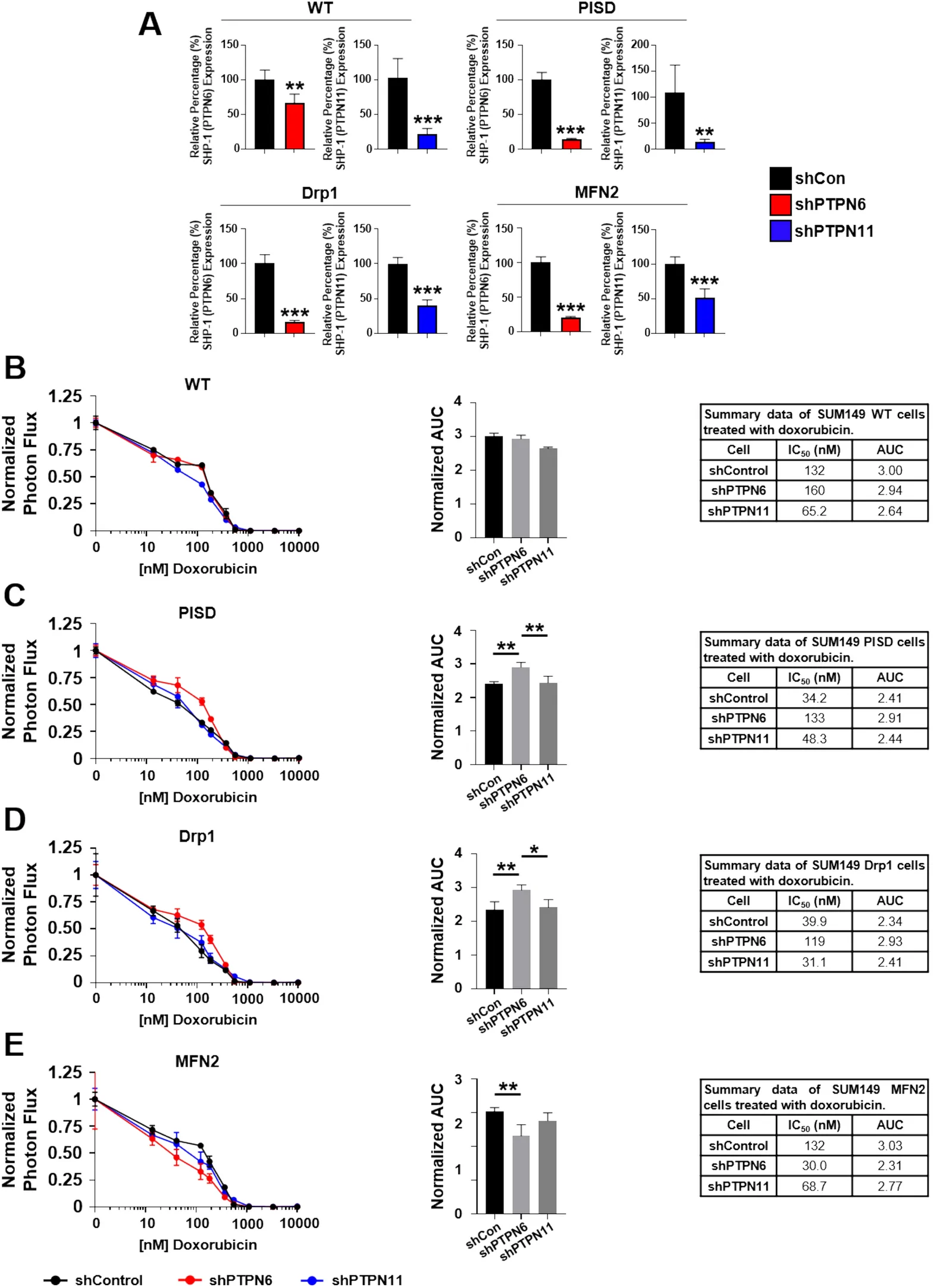
SHP-1 knockdown increases doxorubicin resistance in SUM149 cells with enhanced mitochondrial fission. **A.** qRT-PCR analysis (mean + SEM) of SHP-1 (PTPN6) and SHP-2 (PTPN11) in SUM149 WT, PISD, Drp1, and MFN2 cells (n = 6). Data is relative to SHP-1 and SHP-2 expression in control shRNA (shCon) cells. Data demonstrates that shRNA generates SHP-1 and SHP-2 knockdown in cells. **B-E.** Line graphs show representative mean responses (n = 4) in cellular viability ± SD of SUM149 cells primarily displaying mitochondrial fusion (WT (**B**) and MFN2 (**E**)) or fission (PISD (**C**) and Drp1 (**D**)) with SHP-1 (PTPN6) or SHP-2 (PTPN11) knockdown treated for 72 hours with the commonly used chemotherapeutic, doxorubicin. A decrease along the y-axis represents a decrease in cellular viability versus untreated control. Error bars for SD are smaller than the symbol for some conditions if not evident. Bar graphs demonstrate mean values for area-under-the-curve (AUC) + SEM normalized to untreated control (n = 4) for doxorubicin. Tables on the right summarize the IC_50_ and AUC values for SUM149 cells treated with doxorubicin. All panels represent a distinct, independent experiment. This experiment was repeated at least two other times (n = 3) with similar results. * = *p* < 0.05, ** = *p* < 0.01. *** = *p* < 0.001

To discriminate functions of SHP-1 and SHP-2 in TNBC drug resistance, we utilized shRNA to knockdown SHP-1 or -2 in SUM159 and SUM149 cells. Knockdown of SHP-1 or SHP-2 resulted in a decrease of the corresponding mRNA and protein (Fig 3A, 4A**,** and Supplemental Fig S5) in cells. We also performed a Western blot for STAT3, a known downstream target of these phosphatases [21]. Consistent with our previous work, we found decreased STAT3 phosphorylation in cells that display primarily mitochondrial fission and increased STAT3 phosphorylation in cells that display mitochondrial fusion (Supplemental Fig S6), demonstrating that SHP-1/2 are more active in cells that display enhanced mitochondrial fission. We also found that knockdown of SHP-1/2 rescues this phenotype, as indicated by the increase of STAT3 phosphorylation in knockdown cells. As a preliminary approach to determine effects of SHP-1 or -2 knockdown on TNBC cells, we first determined effects of knockdown on cell proliferation. In line with our previous work [21], control cells that display mitochondrial fusion (WT and MFN2) showed increased cell proliferation compared to control cells that display mitochondrial fission (PISD and Drp1) (Supplemental Fig S7**A**), with no significant differences between cells that display similar mitochondrial dynamics. However, in cells that display enhanced mitochondrial fusion, knockdown of SHP-1 or -2 reduced cell proliferation (Supplemental Fig S7 B-E). Conversely, knockdown of SHP-1 or -2 increased proliferation in cells that display mitochondrial fission (Supplemental Fig S7C-D).

### SHP-1 and -2 knockdown has little effect on paclitaxel sensitivity

We first investigated the effects of SHP-1 or -2 knockdown on paclitaxel resistance. In support of our data above, knockdown of SHP-1 or -2 had minimal impact on paclitaxel sensitivity in both SUM159 (Supplemental Fig S8) and SUM149 (Supplemental Figure S9) cell lines, regardless of mitochondrial dynamics status (WT, PISD, Drp1, or MFN2). More specifically, SUM159 cells showed only minor, context-dependent changes. SHP-1 or -2 knockdown slightly reduced AUC in PISD- or Drp1-expressing cells, but these did not translate into substantial or consistent shifts in IC_50_ (Supplemental Fig S8). SUM149 cells similarly showed that neither AUC nor IC_50_ values differed significantly following SHP-1 or -2 knockdown (Supplemental Fig S9). Overall, these findings indicate that SHP-1 and SHP-2 are not major modulators of paclitaxel response in TNBC cells.

### SHP-1 knockdown drives doxorubicin resistance in cells with enhanced mitochondrial fission

Next, we investigated the effects of SHP-1 and -2 knockdown on doxorubicin sensitivity across SUM159 and SUM149 TNBC cell lines and mitochondrial dynamics states (WT, PISD, Drp1, MFN2). Knockdown of SHP-1 had no significant impact on doxorubicin sensitivity in either SUM159 (Fig 3B) or SUM149 (Fig 4B) WT cells, nor in SUM159 MFN2 cells. However, we do note that increased sensitivity was observed in SUM149 MFN2 cells. In contrast, SHP-1 knockdown reduced doxorubicin sensitivity in PISD- and Drp1-expressing cells for both cell lines (Fig 3B and 4B), suggesting that mitochondrial fission may activate SHP-1 to promote doxorubicin response. We found that the effects on IC_50_ and AUC broadly reflected these trends, with key subgroup comparisons reaching significance. For SHP-2 knockdown, no consistent effect on doxorubicin sensitivity was observed compared to controls in any mitochondrial state, indicating that SHP-2 is not a major modulator of doxorubicin response in our TNBC models.

### SHP-1 knockdown reduces salinomycin sensitivity in cells with enhanced mitochondrial fission

**Fig. 5:**
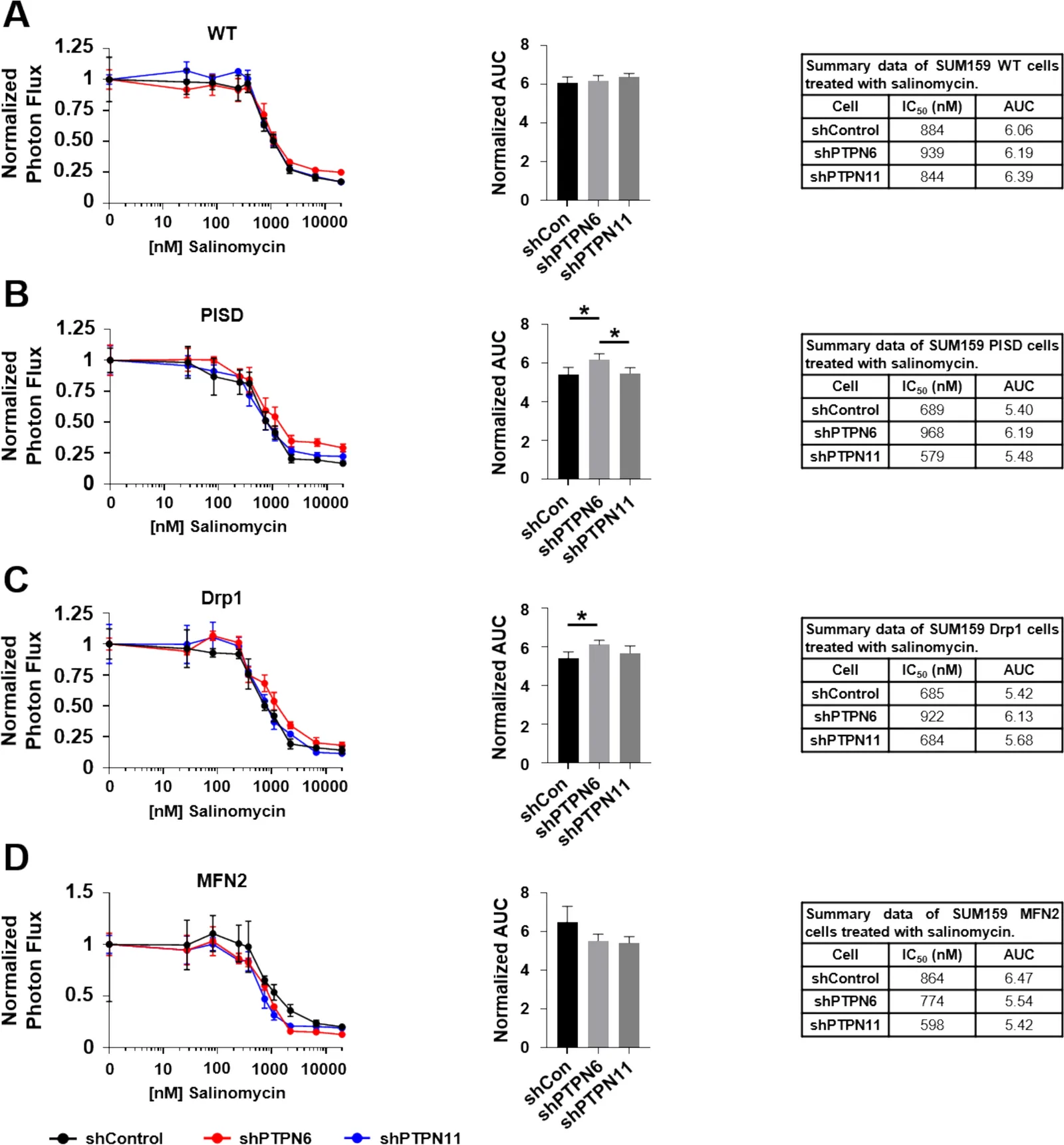
SHP-1 knockdown increases salinomycin resistance in SUM159 cells with enhanced mitochondrial fission. Line graphs show representative mean responses (n = 4) in cellular viability ± SD of SUM159 cells primarily displaying mitochondrial fusion (WT (**A**) and MFN2 (**D**)) or fission (PISD (**B**) and Drp1 (**C**)) with SHP-1 (PTPN6) or SHP-2 (PTPN11) knockdown treated for 72 hours with salinomycin, a cancer stem-like cell targeting compound. A decrease along the y-axis represents a decrease in cellular viability versus untreated control. Error bars for SD are smaller than the symbol for some conditions if not evident. Bar graphs demonstrate mean values for area-under-the-curve (AUC) + SEM normalized to untreated control (n = 4) for salinomycin. Tables on the right summarize the IC_50_ and AUC values for SUM159 cells treated with salinomycin. All panels represent a distinct, independent experiment. This experiment was repeated at least two other times (n = 3) with similar results. * = *p* < 0.05, ** = *p* < 0.01

**Fig. 6:**
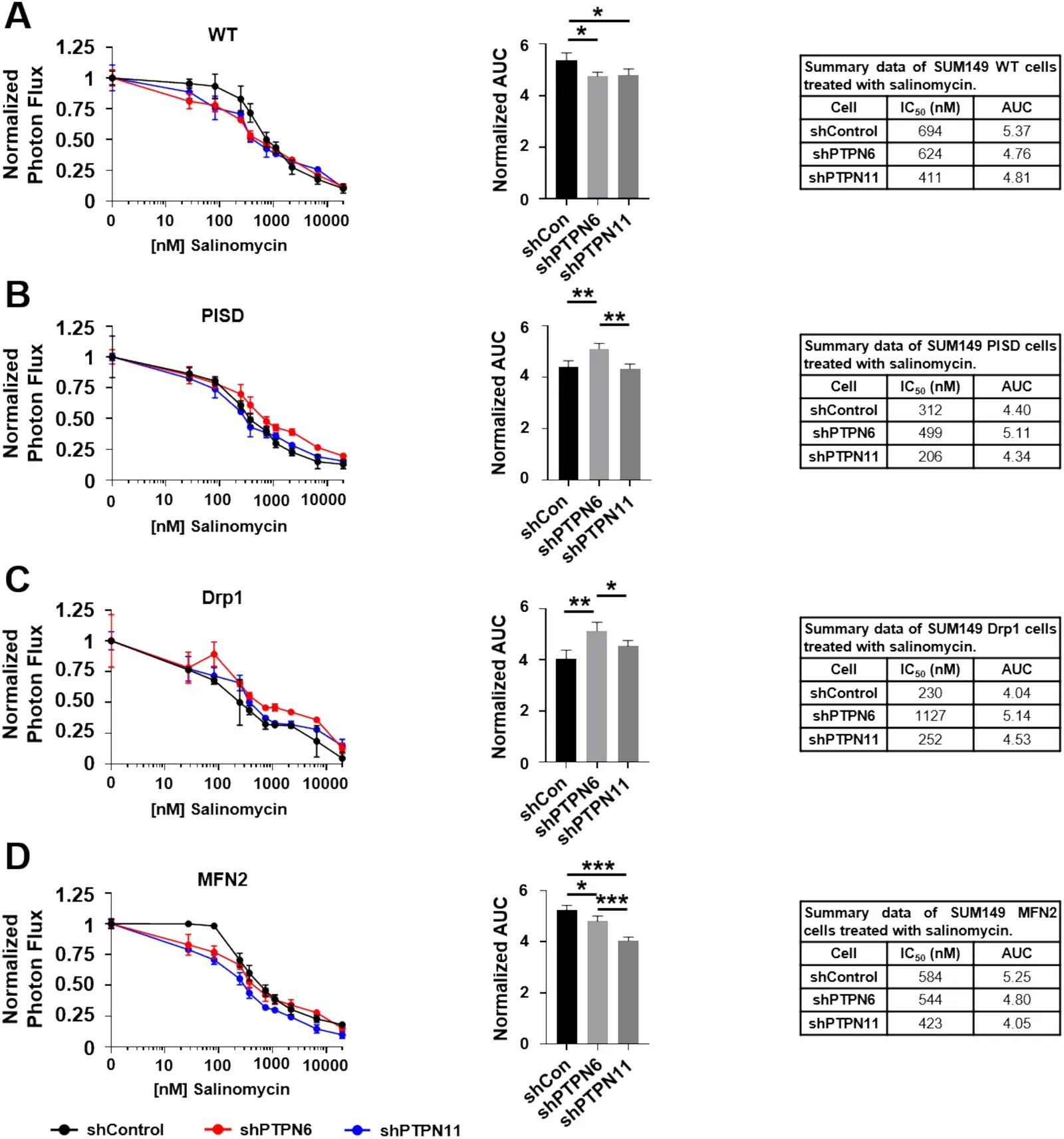
SHP-1 knockdown increases salinomycin resistance in SUM149 cells with enhanced mitochondrial fission. Line graphs show representative mean responses (n = 4) in cellular viability ± SD of SUM149 cells primarily displaying mitochondrial fusion (WT (**A**) and MFN2 (**D**)) or fission (PISD (**B**) and Drp1 (**C**)) with SHP-1 (PTPN6) or SHP-2 (PTPN11) knockdown treated for 72 hours with salinomycin, a cancer stem-like cell targeting compound. A decrease along the y-axis represents a decrease in cellular viability versus untreated control. Error bars for SD are smaller than the symbol for some conditions if not evident. Bar graphs demonstrate mean values for area-under-the-curve (AUC) + SEM normalized to untreated control (n = 4) for salinomycin. Tables on the right summarize the IC_50_ and AUC values for SUM149 cells treated with salinomycin. All panels represent a distinct, independent experiment. This experiment was repeated at least two other times (n = 3) with similar results. * = *p* < 0.05, ** = *p* < 0.01, *** = *p* < 0.001

We also investigated the effects of SHP-1 or -2 knockdown on sensitivity to salinomycin in SUM159 (Fig 5) and SUM149 (Fig 6) TNBC cells. In MFN2-expressing cells, SHP-1 and SHP-2 knockdown produced modest and context-dependent changes in salinomycin response, with some comparisons showing trends toward increased sensitivity (Fig 5D). However, these effects were not consistently significant across both cell lines or across IC_50_ and AUC analyses. WT cells showed similar variable trends, with effects more apparent following SHP-2 knockdown in select comparisons. Conversely, in cells with enhanced mitochondria fission (PISD and Drp1), SHP-1 knockdown reduced salinomycin sensitivity, as reflected by increased IC_50_ and/or AUC values compared to shControl cells. Notably, there were no significant differences in salinomycin response between control and SHP-2 knockdown in PISD or Drp1 cells (Fig 5B-C and 6B-C). Taken together, these results suggest that SHP-1 is an important mediator of salinomycin sensitivity in mitochondrial fission-enriched TNBC cells, whereas SHP-2 effects are more context-dependent and are not consistently observed across models.

## Discussion

Our study demonstrates that mitochondrial dynamics play a critical role in modulating TNBC cell sensitivity to chemotherapeutic agents, acting through the protein-tyrosine phosphatases SHP-1 and -2. We found that TNBC cells with enhanced mitochondrial fission were more sensitive to the first-line drugs doxorubicin and salinomycin, while cells with increased mitochondrial fusion were more resistant to these treatments. Conversely, we found no difference in paclitaxel sensitivity independent of mitochondrial dynamics. Furthermore, inhibition with a SHP-1/2 inhibitor or knockdown of SHP-1 and -2 differentially impacts drug sensitivity, uncovering a nuanced interplay between mitochondrial dynamics and phosphatase signaling in drug resistance.

Tumor-initiating cells (TICs, also known as cancer stem-like cells (CSCs)) have been identified in most solid tumors, including breast cancer, where they are thought to drive tumor initiation, progression, and metastasis [33, 34]. Previous studies, including our own, have demonstrated that TICs possess higher tumorigenicity and metastatic potential than non-TICs in breast cancer models [35–37]. Notably, TICs are implicated in therapy resistance, particularly due to their metabolic adaptability and survival capabilities [38–40]. Recent evidence points to key differences in mitochondria between TICs and non-TICs [31, 41], suggesting that mitochondrial dynamics may underlie the enhanced resilience of TICs. In support of this, we recently reported that enhanced mitochondrial fission reduces TIC phenotypes in TNBC [37]. In this study, we observed that TNBC cells with enhanced mitochondrial fission display increased sensitivity to salinomycin, a drug that selectively targets TICs [32], compared to cells with increased mitochondrial fusion. Although mitochondrial fission has been shown to reduce the abundance and stem-like properties of TICs within TNBC populations, our results indicate that populations with enhanced mitochondrial fission are paradoxically more sensitive to salinomycin. This suggests that mitochondrial fission, while reducing TIC prevalence, may also weaken the metabolic flexibility of residual TICs and other subpopulations, priming them for drug-induced apoptosis. Furthermore, the selective vulnerability driven by mitochondrial fission may extend beyond TICs, enhancing the overall cytotoxic efficacy of agents like salinomycin that target mitochondrial functions disrupted by fission. Therefore, this increased drug sensitivity is likely due to a mitochondrial state-dependent vulnerability rather than to stemness alone. While we observe clear differences in drug sensitivity based upon mitochondrial dynamics, we acknowledge that differences in baseline proliferation rates could influence endpoint viability-based drug response. Because doxorubicin toxicity can be influenced by proliferation rate, the slower growth of mitochondrial fission-enriched populations would be expected to reduce apparent doxorubicin sensitivity. Therefore, our observation that fission-enriched cells display increased doxorubicin sensitivity suggests that fission-associated mitochondrial vulnerabilities and SHP-1 dependent signaling overcome any potential protective effect of reduced proliferation. Future studies using growth rate-corrected drug-response metrics may further clarify the contribution of proliferation rates to these drug effects. Additionally, our findings underscore how even small, mitochondria-driven changes in TIC phenotype or drug sensitivity, whether arising from mutations or altered cellular states, could translate into meaningful differences in clinical outcomes [42].

Another notable observation was the difference in drug sensitivity patterns, where enhanced mitochondrial fission increased TNBC cell sensitivity to doxorubicin and salinomycin, but not paclitaxel. This disparity is likely due to distinct drug mechanisms. Both doxorubicin and salinomycin target key mitochondrial functions, doxorubicin through mitochondrial DNA damage and generation of reactive oxygen species (ROS) [43], and salinomycin by acting as an iron chelator and disturbing mitochondrial metabolism in cancer stem cells [44, 45]. Paclitaxel primarily disrupts microtubule dynamics, a process relatively independent of mitochondrial dynamics, which may explain the lack of differential sensitivity. The fact that salinomycin acts as an iron chelator further suggests that mitochondrial iron metabolism may be pivotal in shaping drug responses, and indeed, iron metabolism has been shown to be important in breast cancer therapy resistance [46–48]. These findings reinforce the concept that the effectiveness of chemotherapeutic agents in TNBC may depend on mitochondrial dynamics.

Our results reveal that SHP-1 serves as the primary effector mediating mitochondrial fission-induced drug sensitivity in TNBC cells. Specifically, knockdown of SHP-1 significantly diminishes sensitivity to doxorubicin and salinomycin in cells with enhanced mitochondrial fission, highlighting the pivotal role of SHP-1 in facilitating drug sensitivity in these cells. In contrast, SHP-2 appears to play a more context-dependent role, where knockdown of SHP-2 alone has a minimal impact on drug sensitivity, suggesting that SHP-2 is not a major regulator of fission-related drug sensitivity. However, pharmacologic inhibition of both SHP-1 and SHP-2 using NSC87877 produced effects that were not fully recapitulated by individual SHP-1 and SHP-2 knockdown, including effects in cells with enhanced mitochondrial fusion. These findings are consistent with possible overlapping or compensatory roles for SHP-2 in a mitochondrial state-dependent manner [49], although additional studies are needed to define the underlying mechanisms. These findings underscore the importance of SHP-1 as a central mediator of mitochondrial fission-driven chemosensitivity, while also pointing to a secondary, compensatory function for SHP-2 in modulating drug sensitivity, particularly in the context of SHP-1 inhibition.

Our findings present new opportunities for TNBC therapy. Notably, our results demonstrate that mitochondrial fission enhances sensitivity of TNBC cells to select chemotherapeutics by activating SHP-1 signaling. This highlights the therapeutic potential of combining standard chemotherapeutics with agents that enforce mitochondrial fission, such as LPE [21, 24], to increase drug efficacy in resistant TNBC. In addition, future studies could evaluate whether direct activation of SHP-1 further enhances chemotherapy response. For example, SC-43 has been reported as a candidate SHP-1 agonist [50], although it was not tested in the present study. Such approaches may be particularly relevant for TIC-enriched, chemoresistant TNBC populations. Ultimately, targeting the mitochondrial fission/SHP-1 axis represents a promising avenue to enhance the success of chemotherapy for TNBC patients.

While our findings uncover important mechanistic links between mitochondrial fission, SHP-1 signaling, and chemosensitivity in TNBC, several limitations warrant consideration. Our results are derived largely from *in vitro* cell line models, which do not fully capture the heterogeneity observed within patient tumors. Moreover, future investigations employing mouse models and patient-derived samples will be critical to validate and extend these findings for clinical translation, especially given the significant roles played by the tumor microenvironment, stromal interactions, and immune modulation.

## Conclusion

Our study demonstrates that enhanced mitochondrial fission increases, while fusion reduces, chemosensitivity in triple-negative breast cancer (TNBC) cells to cytotoxic agents such as doxorubicin and salinomycin. This work also demonstrates SHP-1 as a pivotal effector linking mitochondrial dynamics to drug response, revealing a novel mechanism underlying chemosensitivity in TNBC. The relevance of our findings lies in the identification of the mitochondrial fission/SHP-1 axis as a compelling therapeutic target, where strategies that promote mitochondrial fission or activate SHP-1 may overcome resistance and improve outcomes for patients with aggressive, hard-to-treat breast cancers.

## Supplementary Information

Below is the link to the electronic supplementary material.

Supplementary file1 (PDF 328 KB)

Supplementary file2 (PDF 295 KB)

Supplementary file3 (PDF 226 KB)

Supplementary file4 (PDF 250 KB)

Supplementary file5 (PDF 369 KB)

Supplementary file6 (PDF 225 KB)

Supplementary file7 (PDF 172 KB)

Supplementary file8 (PDF 214 KB)

Supplementary file9 (PDF 201 KB)

Supplementary file10 (PDF 59 KB)

Supplementary file11 (PDF 58 KB)

Supplementary file12 (PDF 58 KB)

Supplementary file13 (PDF 58 KB)

Supplementary file14 (PDF 58 KB)

Supplementary file15 (PDF 58 KB)

## Data Availability

All data has been included in main figures or supplemental information. All data reported in this paper will be shared by the corresponding author contact upon request. This paper does not report original code. Any additional information required to reanalyze the data reported in this paper is available from the corresponding author upon reasonable request.
