# Supplementary material for: Enhanced mitochondrial fission promotes chemosensitivity via SHP-1 activity in triple-negative breast cancer
Source: Mol Biol Rep. 2026 Aug 25;53(1):1459. doi: 10.1007/s11033-026-12615-y (PMC13506572; doi:10.1007/s11033-026-12615-y)
Supplement: Supplementary file 2 [file 11033_2026_12615_MOESM11_ESM.pdf]

Supplemental Table 2. Calculated *p*-values for fraction of mitochondrial fusion in SUM159 cells from data in Supplemental Fig S1 using Tukey's multiple comparisons test.

	WT	WT + LPE	PISD	PISD + Lef	Drp1	MFN2
WT		**	*	n.s.	*	n.s.
WT + LPE			n.s.	*	n.s.	***
PISD				n.s.	n.s.	***
PISD + Lef					*	n.s.
Drp1						***
MFN2						

n.s. = not significant, * = $p < 0.05$, ** = $p < 0.01$, *** = $p < 0.001$.
