# Supplementary material for: Enhanced mitochondrial fission promotes chemosensitivity via SHP-1 activity in triple-negative breast cancer
Source: Mol Biol Rep. 2026 Aug 25;53(1):1459. doi: 10.1007/s11033-026-12615-y (PMC13506572; doi:10.1007/s11033-026-12615-y)
Supplement: Supplementary file 7 [file 11033_2026_12615_MOESM1_ESM.pdf]

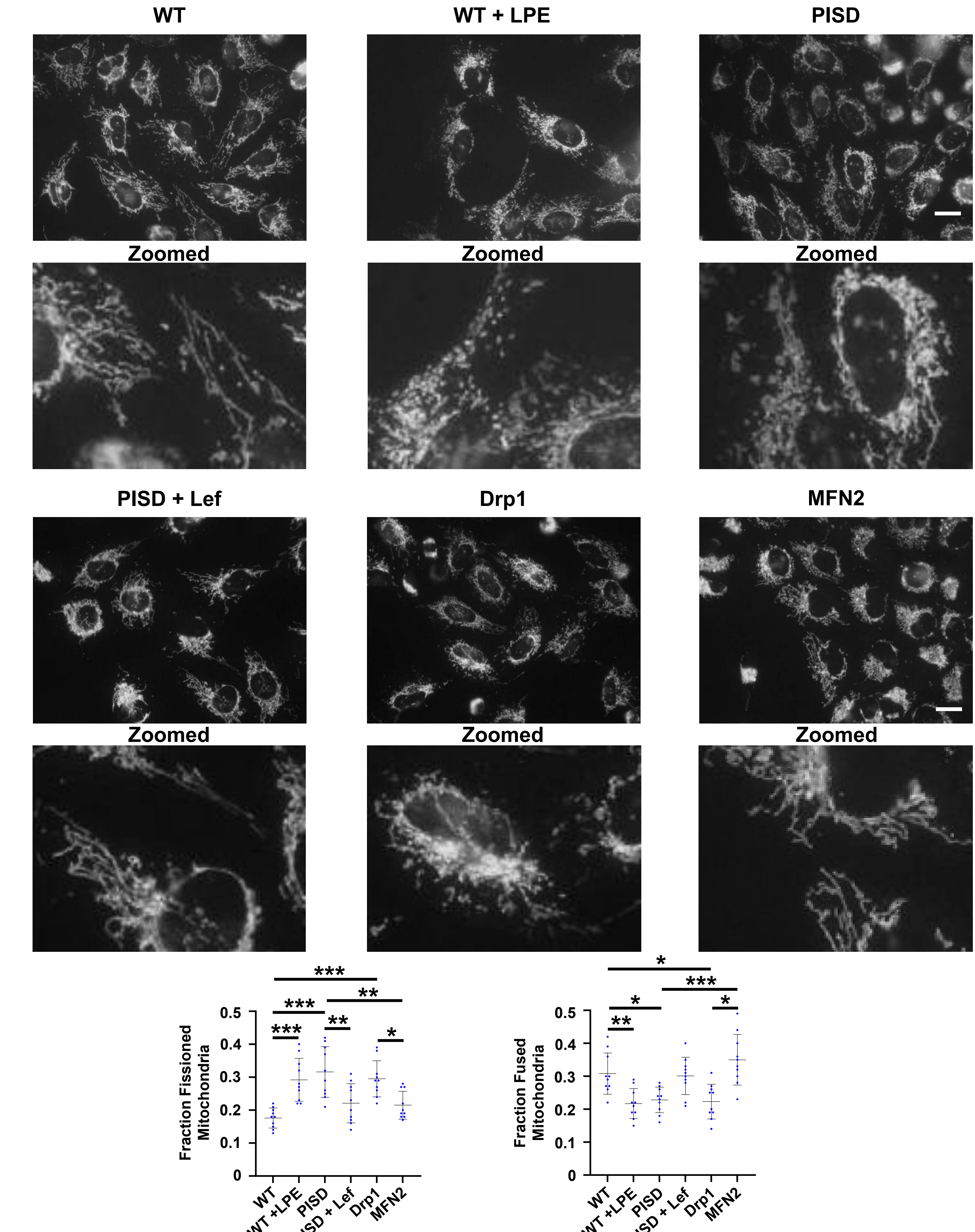

Supplemental Figure S1. Mitochondrial morphology in different SUM159 cell lines. Representative images of WT, WT + LPE, PISD, PISD + Lef, Drp1, and MFN2 stably expressing cells stained with MitoTracker Green. Scale bar is 20 μm. Graphs show average (mean +/- SD) fraction fission (left) or fusion (right) mitochondria in an image in each cell line (n = 10 images). $* = p < 0.05$. $** = p < 0.01$. $*** = p < 0.001$. Prioritized comparisons are shown. All comparisons are shown in Supplemental Tables 1 and 2.
