# Supplementary material for: Enhanced mitochondrial fission promotes chemosensitivity via SHP-1 activity in triple-negative breast cancer
Source: Mol Biol Rep. 2026 Aug 25;53(1):1459. doi: 10.1007/s11033-026-12615-y (PMC13506572; doi:10.1007/s11033-026-12615-y)
Supplement: Supplementary file 8 [file 11033_2026_12615_MOESM2_ESM.pdf]

A

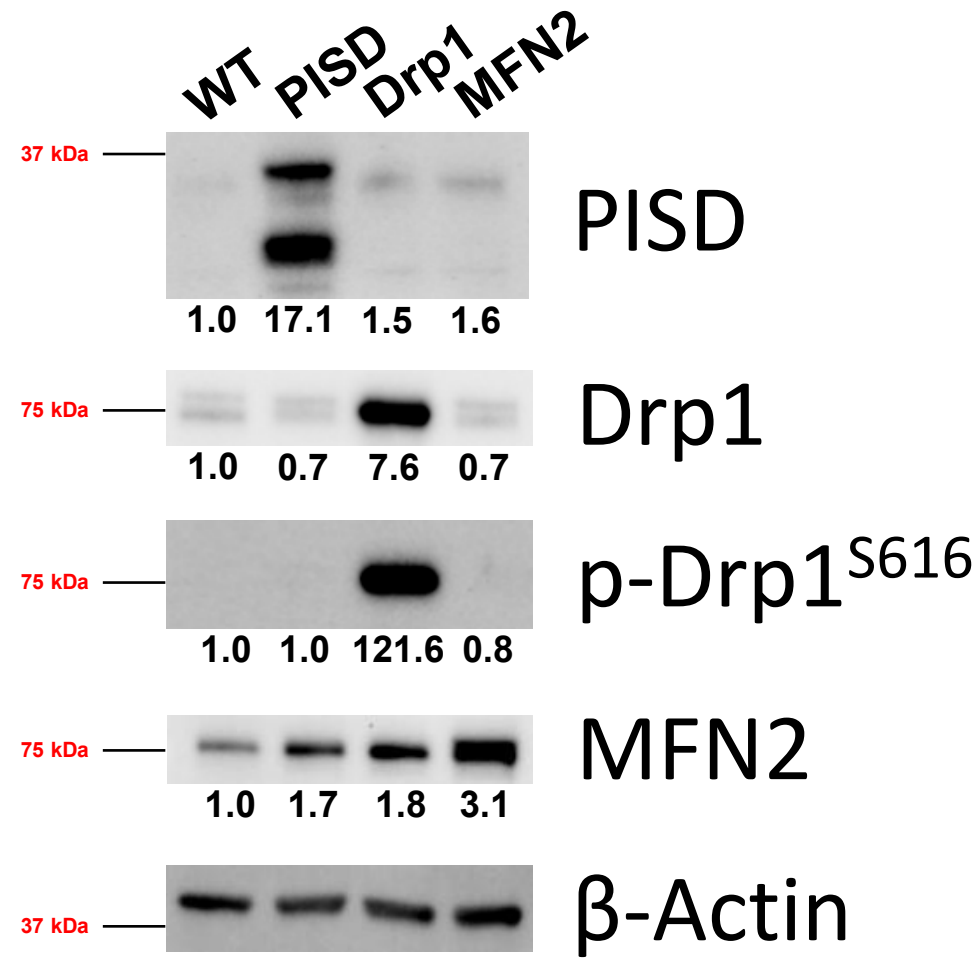

B

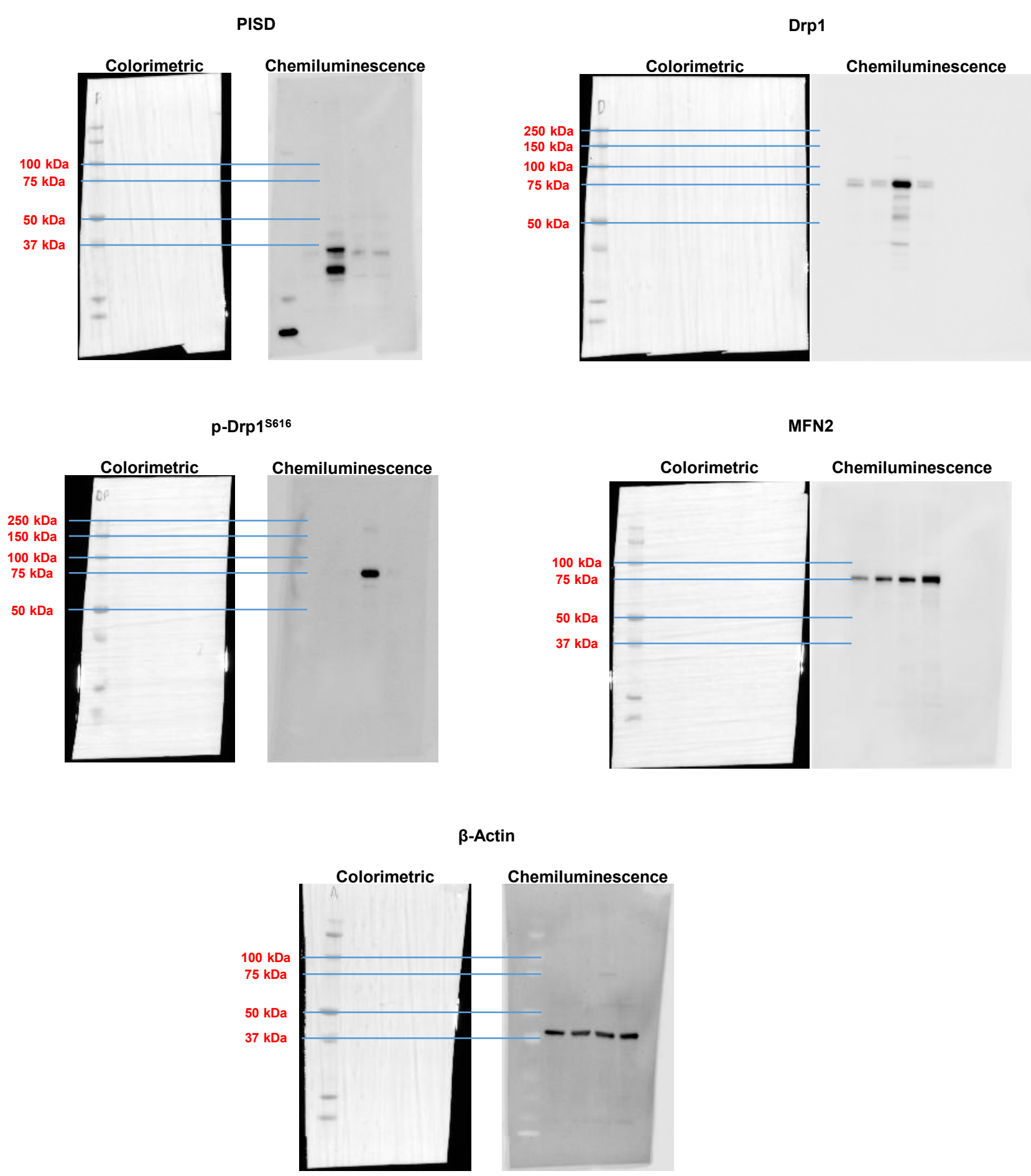

Supplemental Figure S2. Stable expression of proteins that impact mitochondrial morphology in SUM149 cells. Western blot analysis (**A**) and raw Western Blots (**B**) of PISD, Drp1, phosphorylated Drp1 at the known site needed for mitochondrial fission activity (S616), MFN2, and β-actin levels in SUM149 cells. Values under the blots show densitometry values of PISD, Drp1, phosphorylated Drp1, and MFN2 normalized to WT. Data demonstrate that PISD, Drp1, and MFN2 are stably expressed in cells.
