# Supplementary material for: Enhanced mitochondrial fission promotes chemosensitivity via SHP-1 activity in triple-negative breast cancer
Source: Mol Biol Rep. 2026 Aug 25;53(1):1459. doi: 10.1007/s11033-026-12615-y (PMC13506572; doi:10.1007/s11033-026-12615-y)
Supplement: Supplementary file 9 [file 11033_2026_12615_MOESM3_ESM.pdf]

SUM159

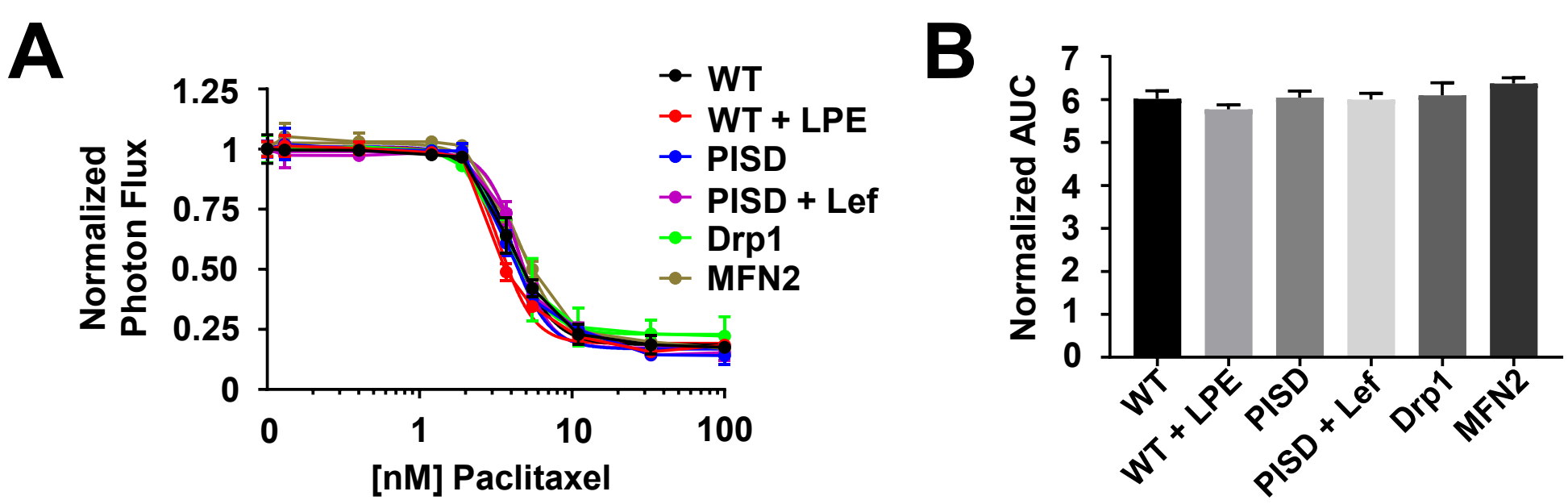

C

Summary data of SUM159 cells treated with paclitaxel.		
Cell	IC_{50} (nM)	AUC
WT	4.09	6.02
WT + LPE	3.37	5.77
PISD	3.92	6.05
PISD + Lef	4.41	6.01
Drp1	3.83	6.1
MFN2	4.54	6.37

SUM149

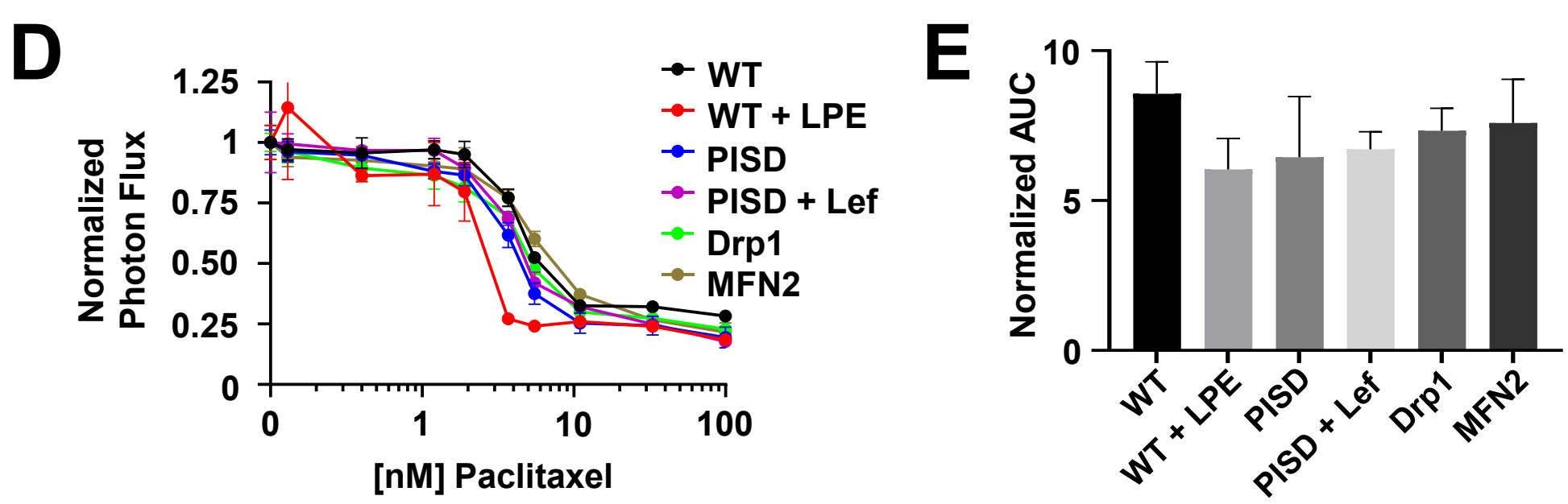

F

Summary data of SUM149 cells treated with paclitaxel.		
Cell	IC_{50} (nM)	AUC
WT	4.53	8.56
WT + LPE	2.29	6.04
PISD	3.51	6.45
PISD + Lef	3.96	6.72
Drp1	4.26	7.33
MFN2	5.79	7.59

Supplemental Figure S3. Mitochondrial dynamics does not significantly impact sensitivity to paclitaxel. Line graphs show representative mean responses (n = 4) in cellular viability ± SD of SUM159 (**A**) and SUM149 (**D**) cells primarily displaying mitochondrial fission (WT + LPE, PISD, and Drp1) or fusion (WT, PISD + Lef, and MFN2) treated for 72 hours with increasing concentrations of the commonly used chemotherapeutic, paclitaxel. A decrease along the y-axis represents a decrease in cellular viability versus untreated control. Error bars for SD are smaller than the symbol for some conditions if not evident. Bar graphs demonstrate mean values for area-under-the-curve (AUC) + SEM normalized to untreated control (n = 4) for paclitaxel (SUM159 (**B**) and SUM149 (**E**)). Tables on the right summarizes the IC_{50} and AUC values for SUM159 and SUM149 cells treated with paclitaxel (SUM159 (**C**) and SUM149 (**F**)).
