# Supplementary material for: Enhanced mitochondrial fission promotes chemosensitivity via SHP-1 activity in triple-negative breast cancer
Source: Mol Biol Rep. 2026 Aug 25;53(1):1459. doi: 10.1007/s11033-026-12615-y (PMC13506572; doi:10.1007/s11033-026-12615-y)
Supplement: Supplementary file 10 [file 11033_2026_12615_MOESM4_ESM.pdf]

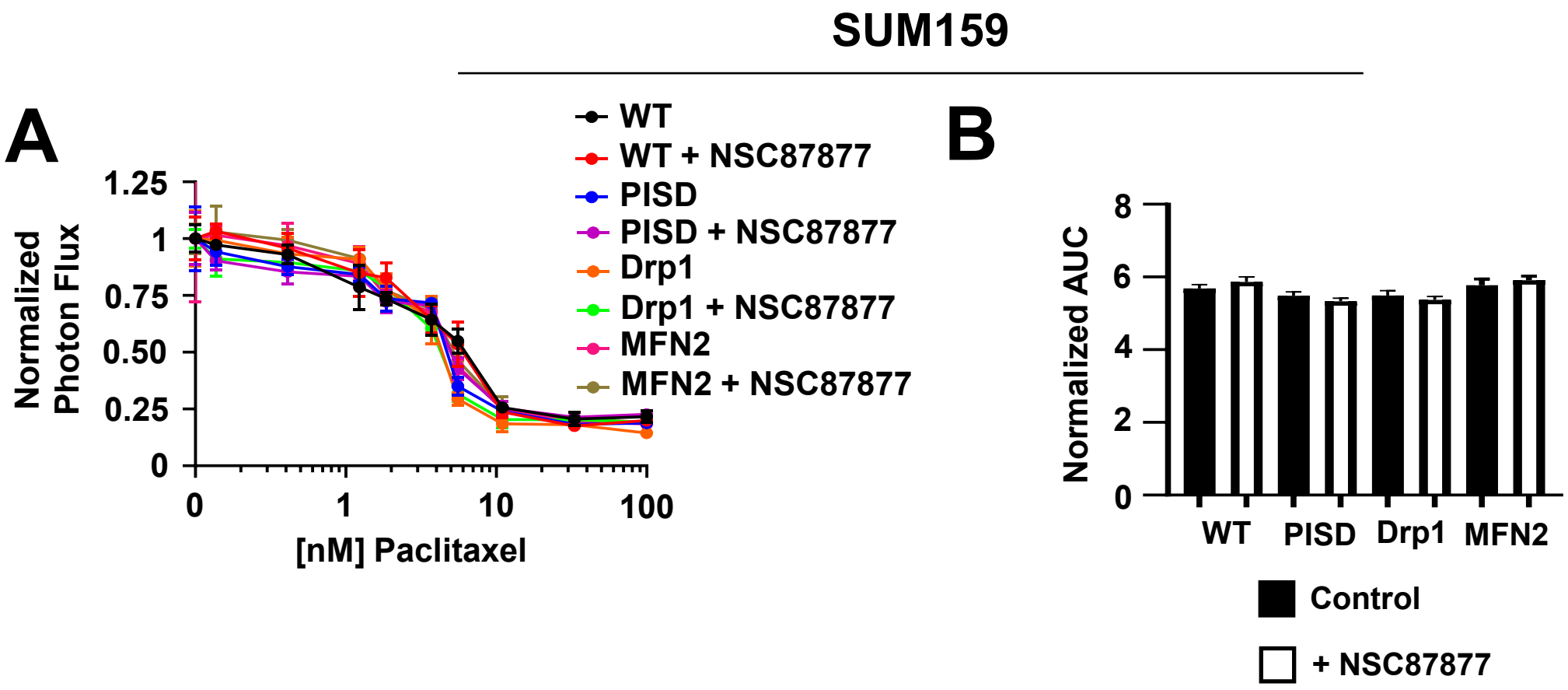

C

Summary data of SUM159 cells treated with paclitaxel.		
Cell	IC_{50} (nM)	AUC
WT	4.04	5.69
WT + NSC87877	4.30	5.86
PISD	4.09	5.48
PISD + NSC87877	4.26	5.33
Drp1	3.66	5.49
Drp1 + NSC87877	3.63	5.37
MFN2	3.86	5.77
MFN2 + NSC87877	3.99	5.91

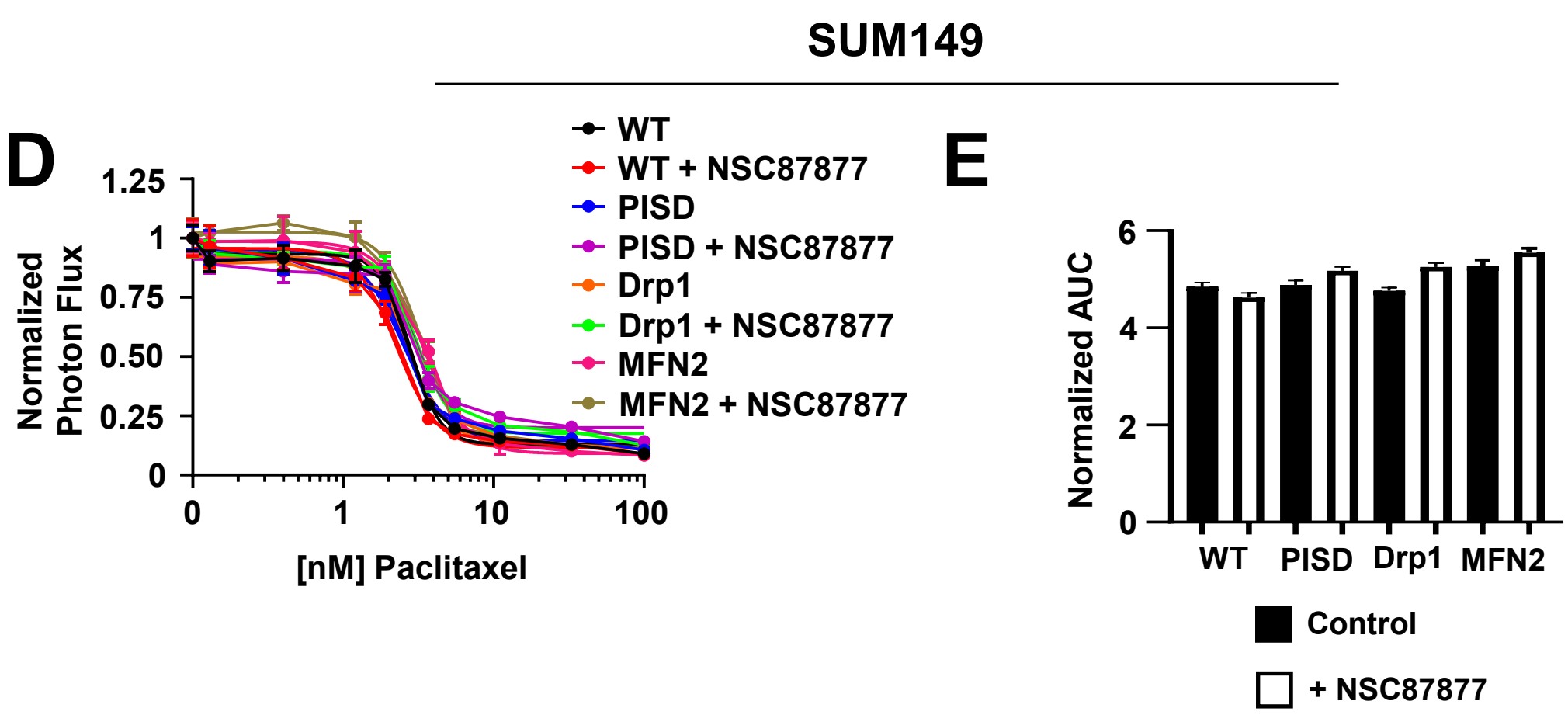

F

Summary data of SUM149 cells treated with paclitaxel.		
Cell	IC_{50} (nM)	AUC
WT	2.81	4.85
WT + NSC87877	2.29	4.62
PISD	2.51	4.88
PISD + NSC87877	3.05	5.17
Drp1	2.68	4.76
Drp1 + NSC87877	3.10	5.25
MFN2	3.42	5.27
MFN2 + NSC87877	3.29	5.54

Supplemental Figure S4. SHP1/2 inhibition does not significantly impact sensitivity to paclitaxel. Line graphs show representative mean responses (n = 4) in cellular viability ± SD of SUM159 (**A**) and SUM149 (**D**) cells primarily displaying mitochondrial fission (PISD and Drp1) or fusion (WT and MFN2) treated with or without the SHP-1/2 inhibitor NSC87877 treated for 72 hours with the commonly used chemotherapeutic, paclitaxel. A decrease along the y-axis represents a decrease in cellular viability versus untreated control. Error bars for SD are smaller than the symbol for some conditions if not evident. Bar graphs demonstrate mean values for area-under-the-curve (AUC) + SEM normalized to untreated control (n = 4) for paclitaxel (SUM159 (**B**) and SUM149 (**E**)). Tables on the right summarizes the IC_{50} and AUC values for SUM159 and SUM149 cells treated with paclitaxel (SUM159 (**C**) and SUM149 (**F**)). * = $p < 0.05$, ** = $p < 0.01$.
