# Supplementary material for: Enhanced mitochondrial fission promotes chemosensitivity via SHP-1 activity in triple-negative breast cancer
Source: Mol Biol Rep. 2026 Aug 25;53(1):1459. doi: 10.1007/s11033-026-12615-y (PMC13506572; doi:10.1007/s11033-026-12615-y)
Supplement: Supplementary file 11 [file 11033_2026_12615_MOESM5_ESM.pdf]

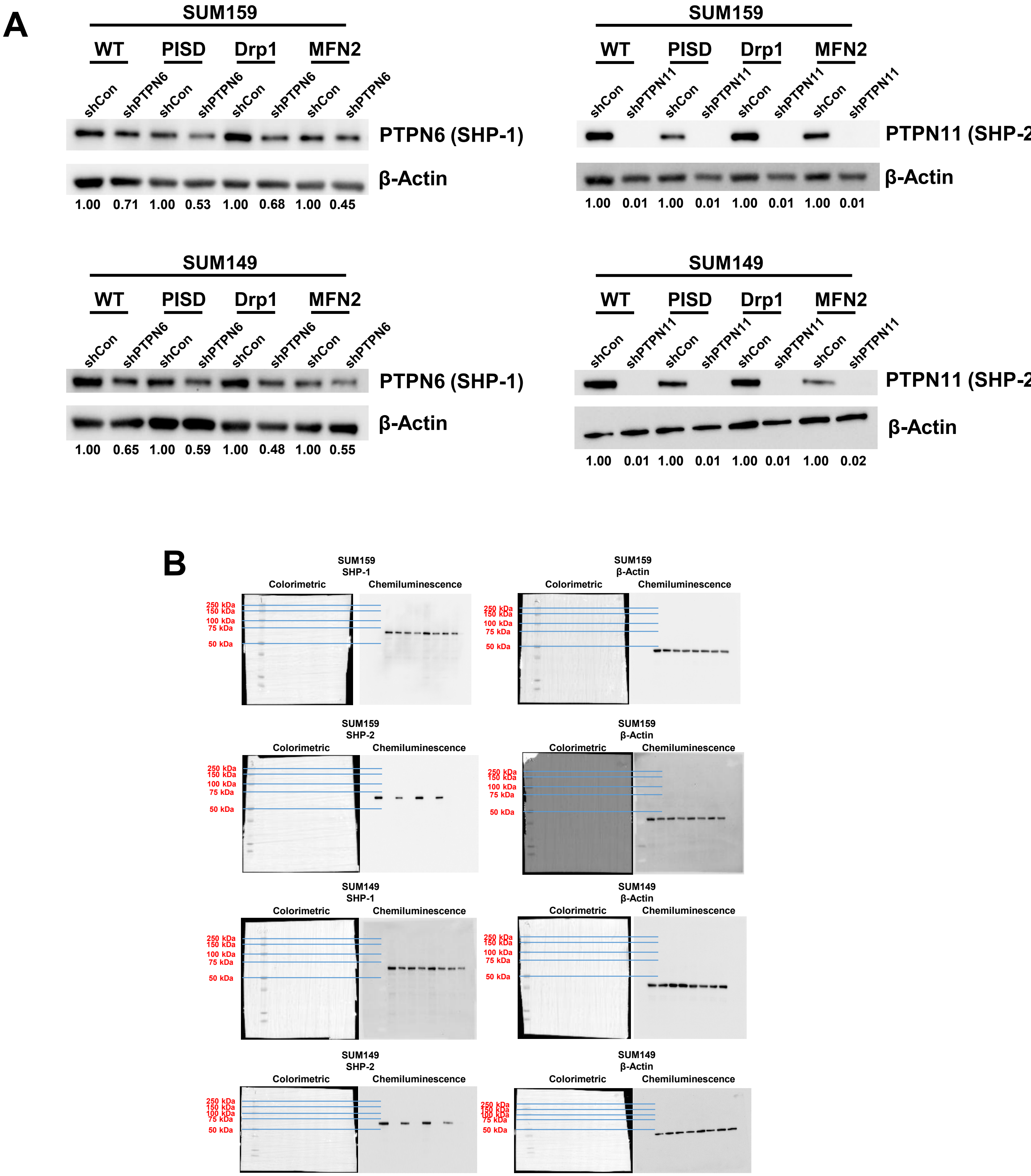

Supplemental Figure S5. SHP-1 and SHP-2 knockdown in SUM159 and SUM149 cells. Western blot analysis (**A**) and raw Western Blots (**B**) of SHP-1/2 and β-actin levels in SUM159 and SUM149 cells. Values under the blots show densitometry values of SHP-1/2 relative to β-actin and normalized to control cells (shCon in WT, PISD, Drp1, or MFN2). Data demonstrate that SHP-1/2 are knocked down in these cells.
