# Supplementary material for: Enhanced mitochondrial fission promotes chemosensitivity via SHP-1 activity in triple-negative breast cancer
Source: Mol Biol Rep. 2026 Aug 25;53(1):1459. doi: 10.1007/s11033-026-12615-y (PMC13506572; doi:10.1007/s11033-026-12615-y)
Supplement: Supplementary file 12 [file 11033_2026_12615_MOESM6_ESM.pdf]

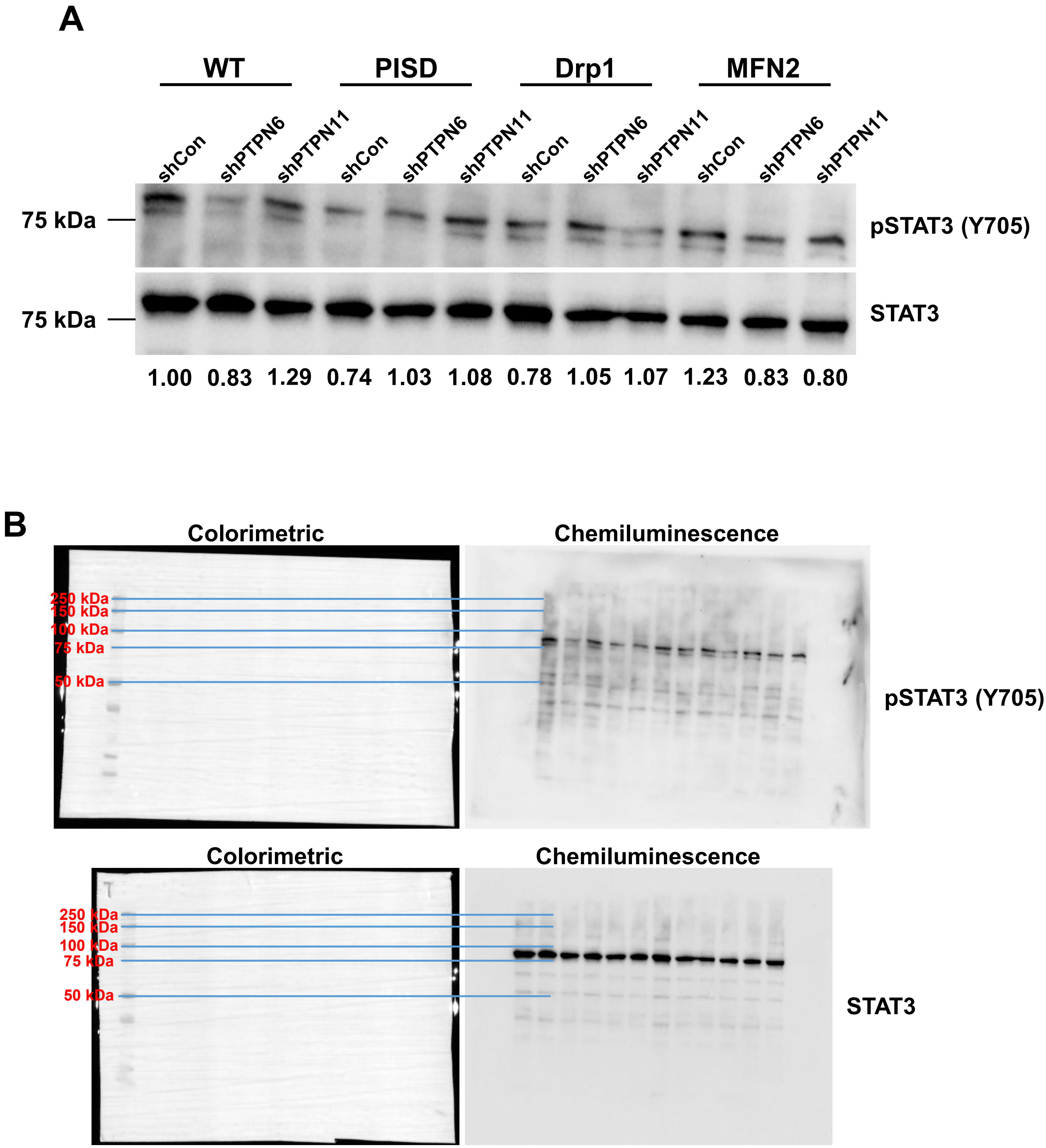

Supplemental Figure S6. Mitochondrial fission reduces, and SHP-1/2 knockdown increase, phosphorylation of STAT3. Western blot analysis (**A**) and raw Western Blots (**B**) of phosphorylated STAT3 at the SHP-1/2 known dephosphorylation site (Y705) and total STAT3 levels in SUM159 cells. Values under the blots show densitometry values of phosphorylated/total STAT3 normalized to WT. Data demonstrate that SHP-1/2 are more activated in cells that display enhanced mitochondrial fission, and that knockdown of SHP-1/2 increases STAT3 phosphorylation in these cells.
