# Supplementary material for: Enhanced mitochondrial fission promotes chemosensitivity via SHP-1 activity in triple-negative breast cancer
Source: Mol Biol Rep. 2026 Aug 25;53(1):1459. doi: 10.1007/s11033-026-12615-y (PMC13506572; doi:10.1007/s11033-026-12615-y)
Supplement: Supplementary file 13 [file 11033_2026_12615_MOESM7_ESM.pdf]

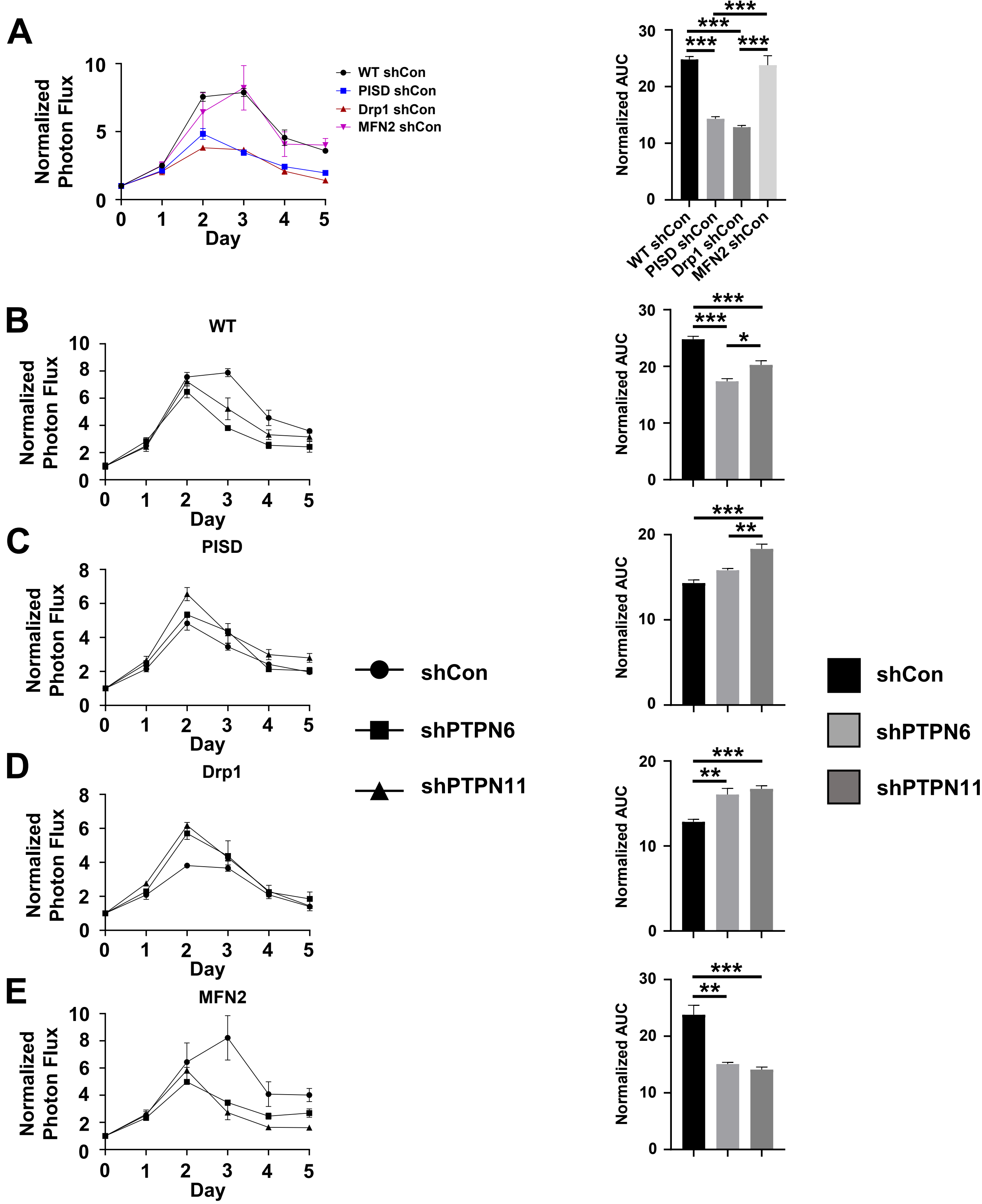


**Supplemental Figure S7. shRNA knockdown of SHP1 and SHP2 in cells that stably express proteins that impact mitochondrial morphology alters cell growth.** Relative growth (line graphs (**left**)) and area-under-the-curve (AUC, **right**) of SUM159 cells that display mitochondrial fusion (WT and MFN2) or fission (PISD and Drp1). Line graphs show mean values ± SD for bioluminescence photon flux at time shown (Days 1-5) relative to initial bioluminescence photon flux after seeding (Day 0) (n = 4). Bar graphs show AUC + SEM of line graphs. * = $p < 0.05$, ** = $p < 0.01$, *** = $p < 0.001$.
