# Supplementary material for: Enhanced mitochondrial fission promotes chemosensitivity via SHP-1 activity in triple-negative breast cancer
Source: Mol Biol Rep. 2026 Aug 25;53(1):1459. doi: 10.1007/s11033-026-12615-y (PMC13506572; doi:10.1007/s11033-026-12615-y)
Supplement: Supplementary file 14 [file 11033_2026_12615_MOESM8_ESM.pdf]

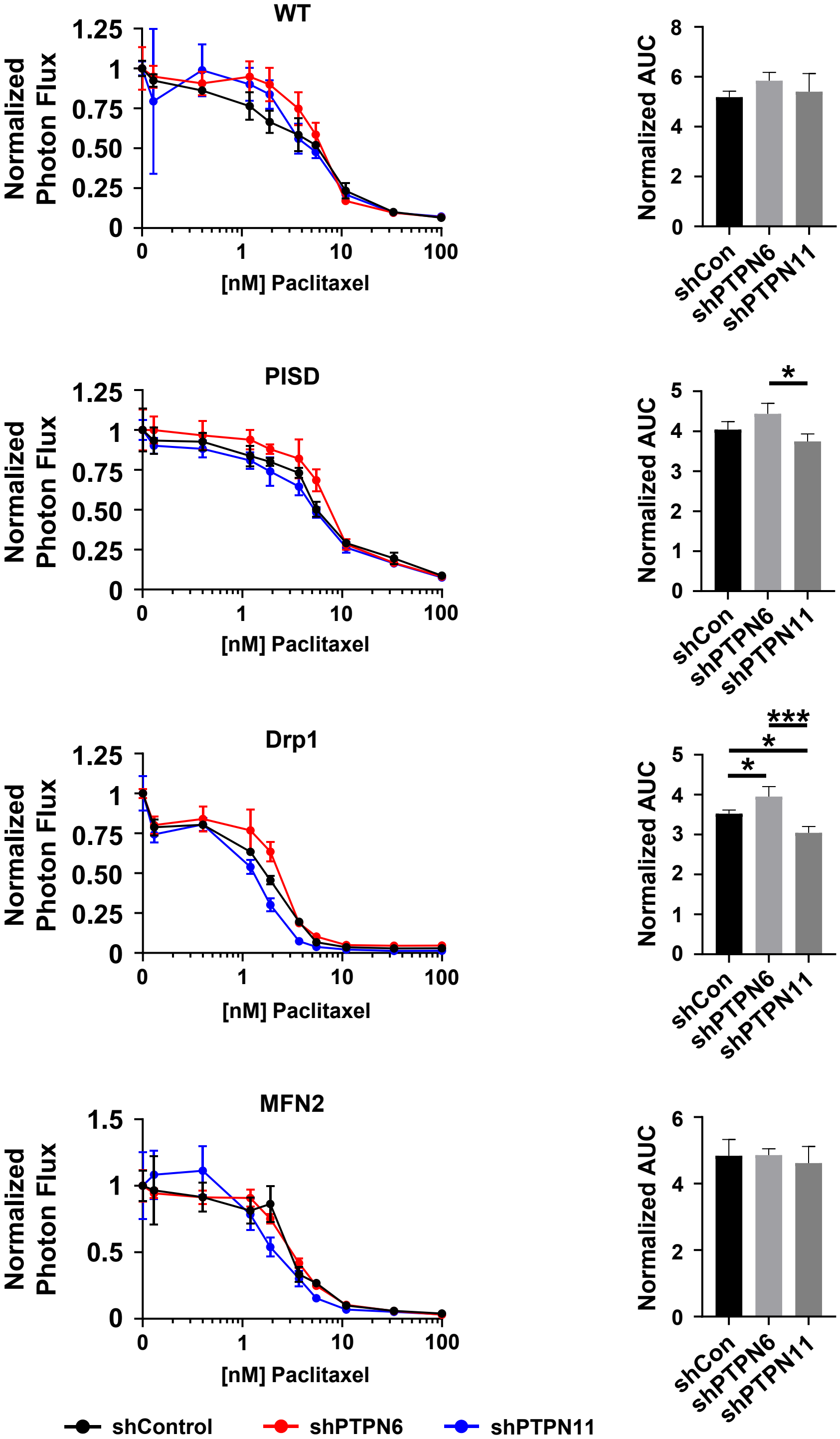

Summary data of SUM159 WT cells treated with paclitaxel.

Cell	IC_{50} (nM)	AUC
shControl	7.07	5.19
shPTPN6	7.11	5.84
shPTPN11	6.80	5.40

Summary data of SUM159 PISD cells treated with paclitaxel.

Cell	IC_{50} (nM)	AUC
shControl	5.18	4.04
shPTPN6	5.44	4.44
shPTPN11	5.21	3.74

Summary data of SUM159 Drp1 cells treated with paclitaxel.

Cell	IC_{50} (nM)	AUC
shControl	4.86	3.52
shPTPN6	5.35	3.95
shPTPN11	4.36	3.04

Summary data of SUM159 MFN2 cells treated with paclitaxel.

Cell	IC_{50} (nM)	AUC
shControl	5.78	4.84
shPTPN6	5.86	4.86
shPTPN11	4.97	4.62

Supplemental Figure S8. SHP-1 knockdown has inconsistent effects on paclitaxel resistance in SUM159 cells. Line graphs show representative mean responses (n = 4) in cellular viability ± SD of SUM159 cells primarily displaying mitochondrial fission (PISD and Drp1) or fusion (WT and MFN2) with SHP-1 (PTPN6) or SHP-2 (PTPN11) knockdown treated for 72 hours with paclitaxel. A decrease along the y-axis represents a decrease in cellular viability versus untreated control. Error bars for SD are smaller than the symbol for some conditions if not evident. Bar graphs demonstrate mean values for area-under-the-curve (AUC) + SEM normalized to untreated control (n = 4) for paclitaxel. Tables on the right summarizes the IC_{50} and AUC values for SUM159 cells treated with paclitaxel. * = $p < 0.05$, *** = $p < 0.001$.
