# Supplementary material for: Enhanced mitochondrial fission promotes chemosensitivity via SHP-1 activity in triple-negative breast cancer
Source: Mol Biol Rep. 2026 Aug 25;53(1):1459. doi: 10.1007/s11033-026-12615-y (PMC13506572; doi:10.1007/s11033-026-12615-y)
Supplement: Supplementary file 15 [file 11033_2026_12615_MOESM9_ESM.pdf]

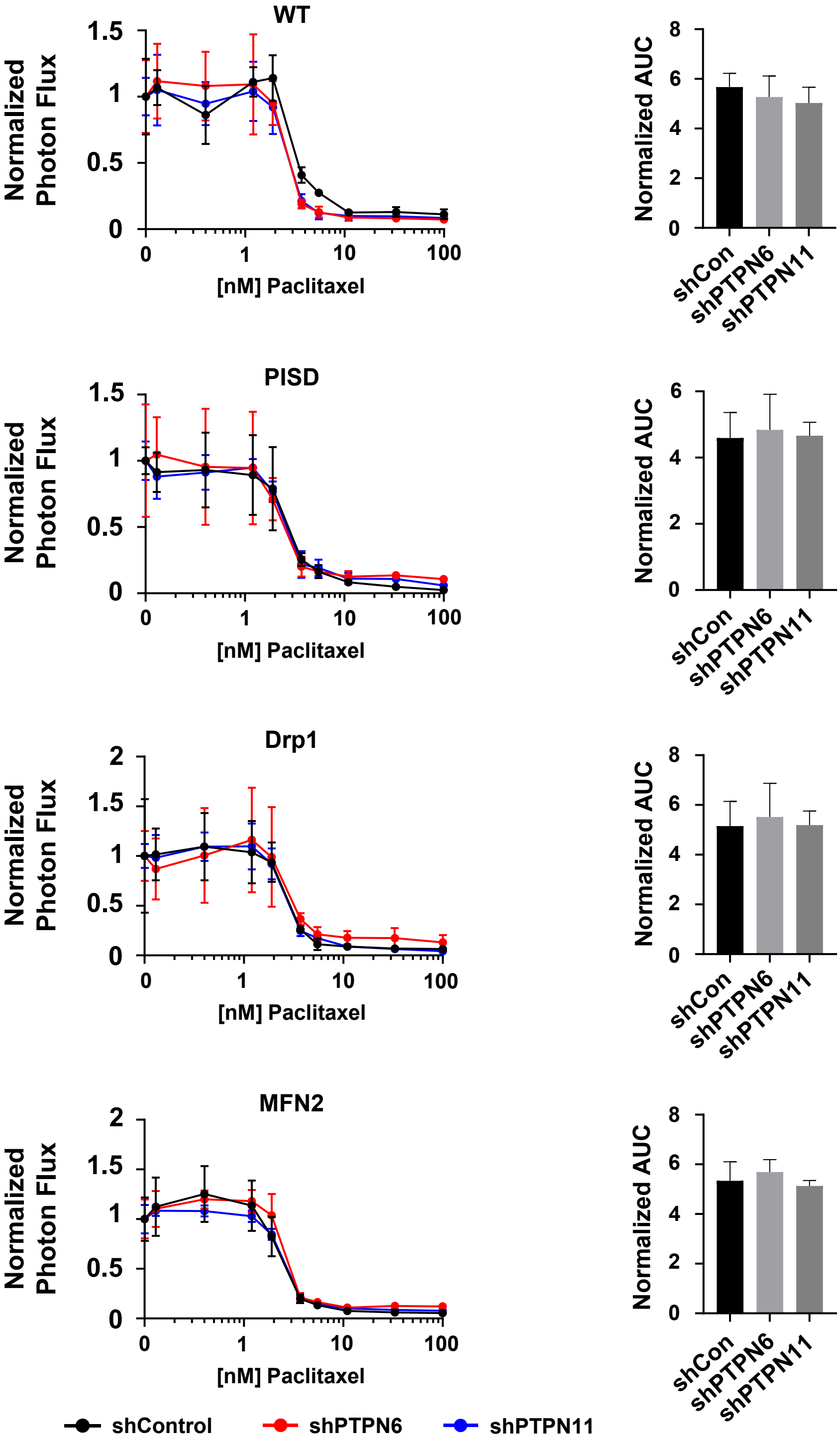

Summary data of SUM149 WT cells treated with paclitaxel.		
**Cell**	**IC_{50} (nM)**	**AUC**
**shControl**	3.35	5.67
**shPTPN6**	2.61	5.27
**shPTPN11**	2.72	5.03

Summary data of SUM149 PISD cells treated with paclitaxel.		
**Cell**	**IC_{50} (nM)**	**AUC**
**shControl**	2.77	4.59
**shPTPN6**	2.22	4.84
**shPTPN11**	2.55	4.66

Summary data of SUM149 Drp1 cells treated with paclitaxel.		
**Cell**	**IC_{50} (nM)**	**AUC**
**shControl**	2.83	5.15
**shPTPN6**	3.15	5.52
**shPTPN11**	2.78	5.19

Summary data of SUM149 MFN2 cells treated with paclitaxel.		
**Cell**	**IC_{50} (nM)**	**AUC**
**shControl**	3.34	5.33
**shPTPN6**	3.63	5.69
**shPTPN11**	3.49	5.13

Supplemental Figure S9. SHP-1 nor SHP-2 impacts paclitaxel sensitivity in SUM149 cells. Line graphs show representative mean responses (n = 4) in cellular viability ± SD of SUM149 cells primarily displaying mitochondrial fission (PISD and Drp1) or fusion (WT and MFN2) with SHP-1 (PTPN6) or SHP-2 (PTPN11) knockdown treated for 72 hours with paclitaxel. A decrease along the y-axis represents a decrease in cellular viability versus untreated control. Error bars for SD are smaller than the symbol for some conditions if not evident. Bar graphs demonstrate mean values for area-under-the-curve (AUC) + SEM normalized to untreated control (n = 4) for paclitaxel. Tables on the right summarizes the IC_{50} and AUC values for SUM149 cells treated with paclitaxel.
